# Supplementary material for: Synthesis and Evaluation of Some New 4H-Pyran Derivatives as Antioxidant, Antibacterial and Anti-HCT-116 Cells of CRC, with Molecular Docking, Antiproliferative, Apoptotic and ADME Investigations
Source: Pharmaceuticals (Basel). 2022 Jul 19;15(7):891. doi: 10.3390/ph15070891 (PMC9317316; doi:10.3390/ph15070891)
Supplement: Supplementary file 1 [file pharmaceuticals-15-00891-s001.zip › pharmaceuticals-1758306-supplementary.PDF]

Synthesis and evaluation of some 4*H*-pyran derivatives as antioxidant, antibacterial and anti-HCT-116 cells of CRC. Molecular docking, quantitative measurement of CDK2 and real time PCR determination of the expression profiles of Caspase-3 and CDK genes

Nahed N. E. El-Sayed, Magdi E. A. Zaki, Sami A. Al-Hussain, Abir Ben Bacha, Malika Berredjem, Vijay H. Masand, Zainab M. Almarhoon, Hanaa S. Omar

Representative spectra

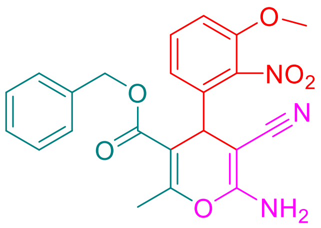

Compound 4c

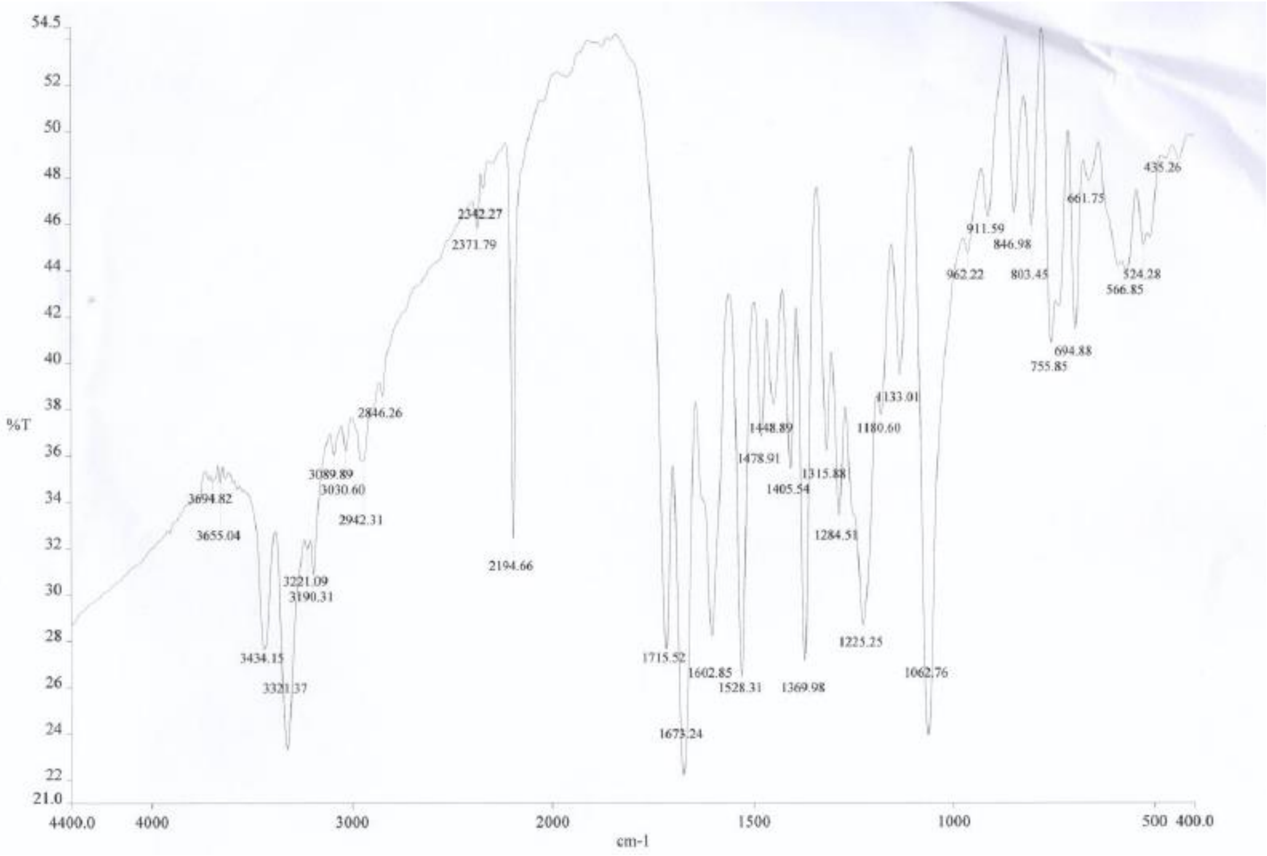
%T
cm-1
54.5
52
50
48
46
44
42
40
38
36
34
32
30
28
26
24
22
21.0
4400.0
4000
3000
2000
1500
1000
500
400.0
3694.82
3655.04
3434.15
3321.37
3221.09
3190.31
3089.89
3030.60
2942.31
2846.26
2342.27
2371.79
2194.66
1715.52
1673.24
1602.85
1528.31
1478.91
1448.89
1405.54
1369.98
1315.88
1284.51
1225.25
1180.60
1133.01
1062.76
962.22
911.59
846.98
803.45
755.85
694.88
661.75
566.85
524.28
435.26

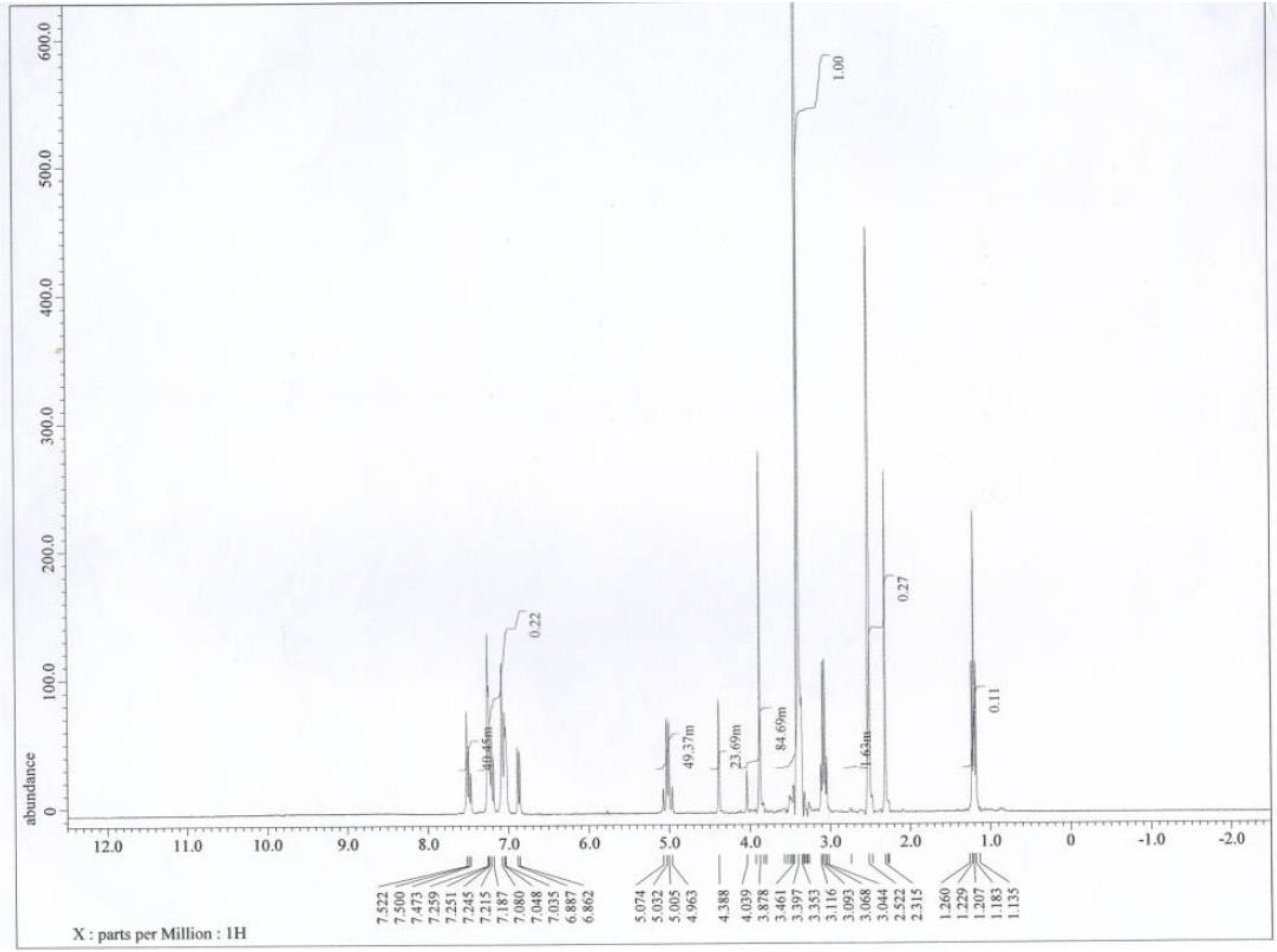
abundance
X : parts per Million : 1H
7.522
7.500
7.473
7.259
7.251
7.245
7.215
7.187
7.080
7.048
7.035
6.887
6.862
5.074
5.032
5.005
4.963
4.388
4.039
3.878
3.461
3.397
3.353
3.116
3.093
3.068
3.044
2.522
2.315
1.260
1.229
1.207
1.183
1.135
1.00
0.22
0.27
0.11
40.45m
49.37m
23.69m
84.69m
1.63m

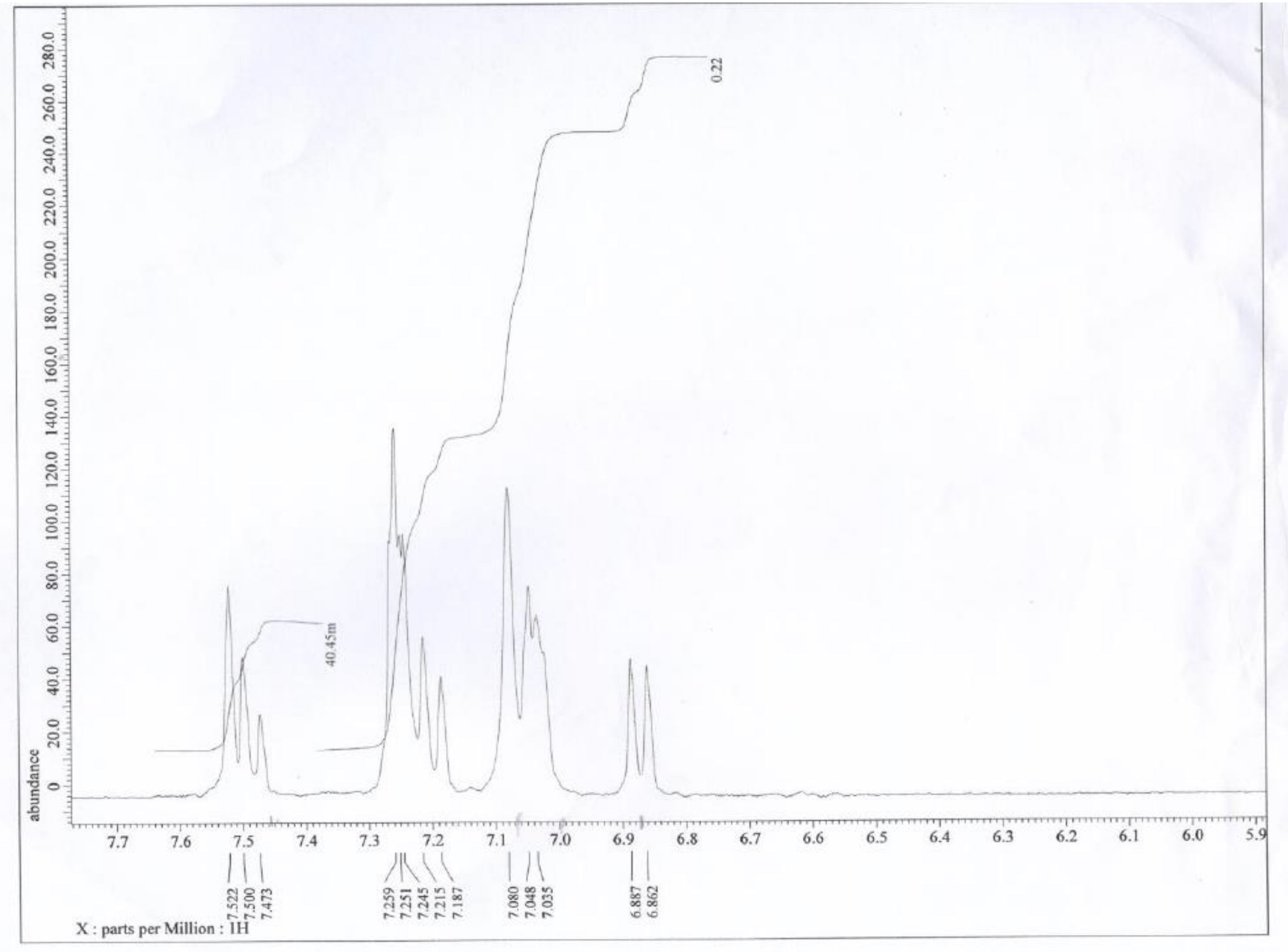
abundance
0
20.0
40.0
60.0
80.0
100.0
120.0
140.0
160.0
180.0
200.0
220.0
240.0
260.0
280.0
40.45m
0.22
7.7
7.6
7.5
7.4
7.3
7.2
7.1
7.0
6.9
6.8
6.7
6.6
6.5
6.4
6.3
6.2
6.1
6.0
5.9
7.522
7.500
7.473
7.259
7.251
7.245
7.215
7.187
7.080
7.048
7.035
6.887
6.862
X : parts per Million : 1H

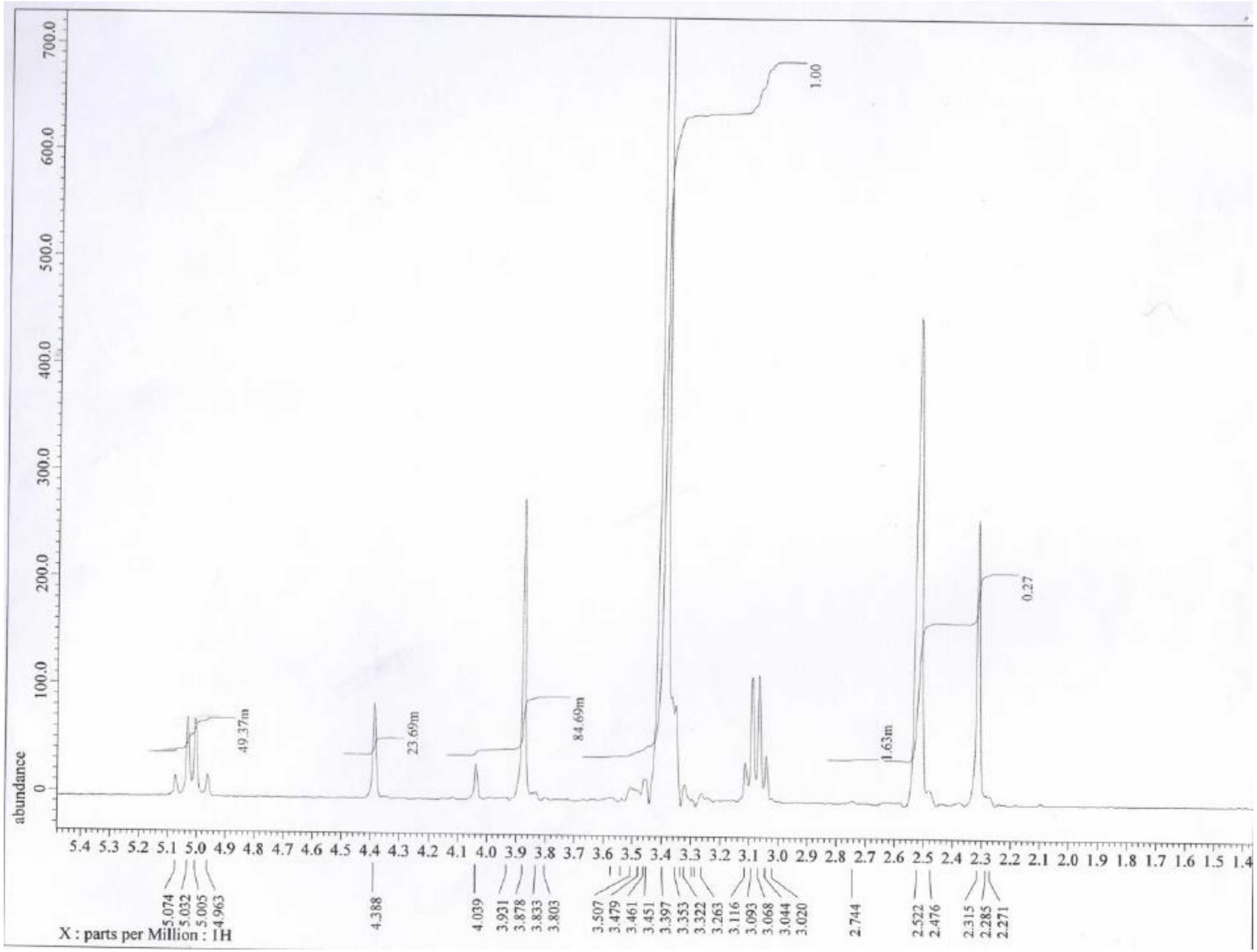
abundance
X : parts per Million : 1H
49.37m
23.69m
84.69m
1.00
1.63m
0.27
5.074 5.032 5.005 4.963
4.388
4.039 3.931 3.878 3.833 3.803
3.507 3.479 3.461 3.451 3.397 3.353 3.322 3.263 3.116 3.093 3.068 3.044 3.020
2.744
2.522 2.476
2.315 2.285 2.271

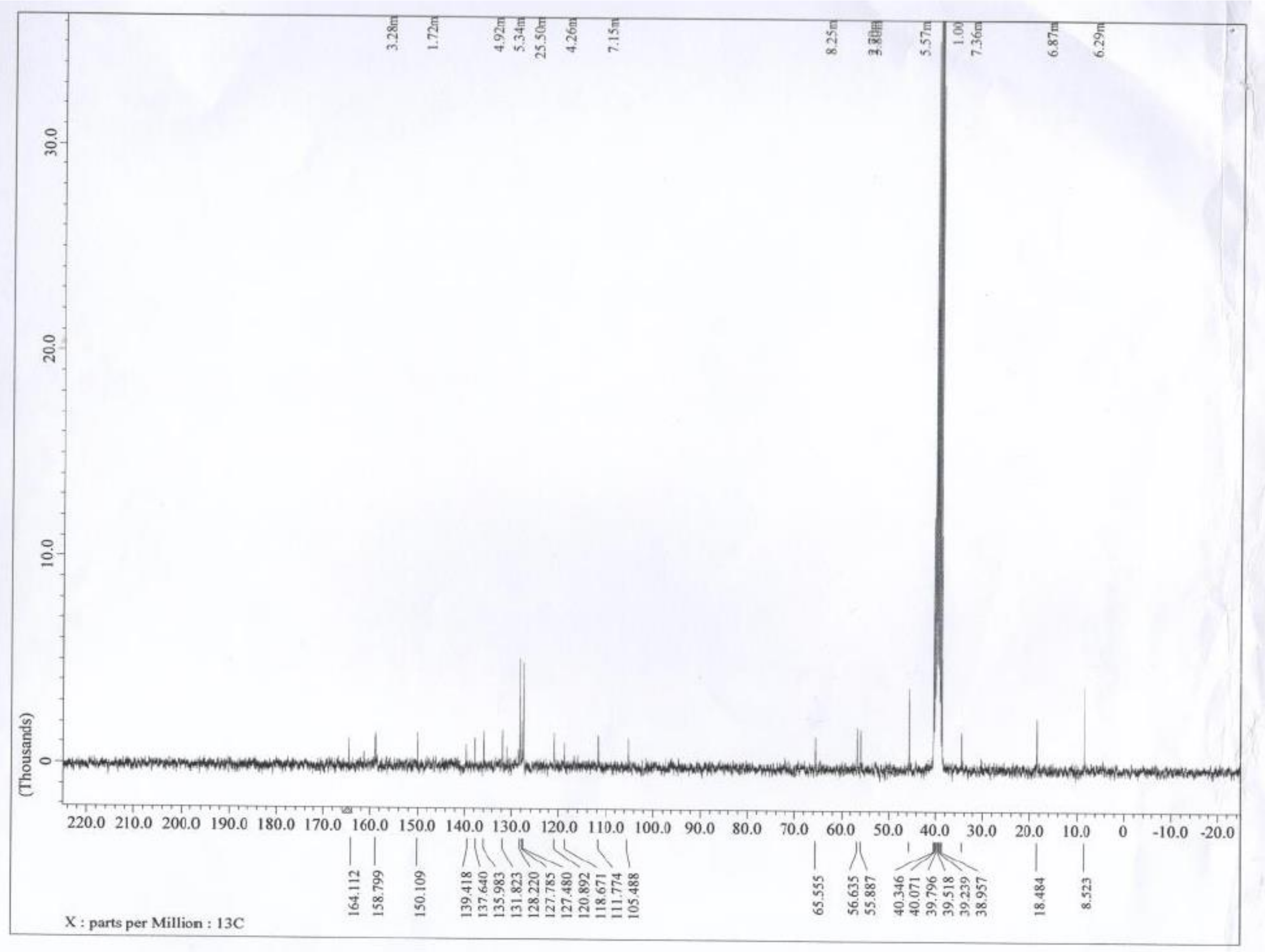

(Thousands)
30.0
20.0
10.0
0
220.0 210.0 200.0 190.0 180.0 170.0 160.0 150.0 140.0 130.0 120.0 110.0 100.0 90.0 80.0 70.0 60.0 50.0 40.0 30.0 20.0 10.0 0 -10.0 -20.0
164.112
158.799
150.109
139.418
137.640
135.983
131.823
128.220
127.785
127.480
120.892
118.671
111.774
105.488
65.555
56.635
55.887
40.346
40.071
39.796
39.518
39.239
38.957
18.484
8.523
X : parts per Million : 13C

Line#:1 R.Time:6.5(Scan#:776)
MassPeaks:290
RawMode:Single 6.5(776) BasePeak:91(2876938)
BG Mode:None Group 1 - Event 1

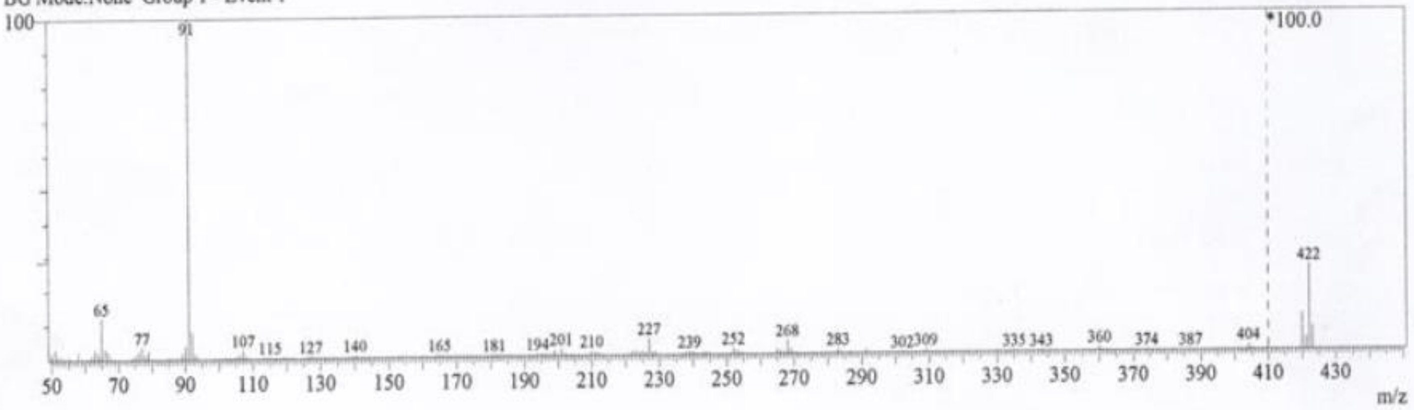

Mass Table
Line#:1 R.Time:6.5(Scan#:776)
MassPeaks:290
RawMode:Single 6.5(776) BasePeak:91(2876938)
BG Mode:None Group 1 - Event 1

#	m/z	Abs. In	Rel. Int.	#	m/z	Abs. In	Rel. Int.	#	m/z	Abs. In	Rel. Int.
1	50.00	42142	1.46	4	53.00	25657	0.89	7	56.00	2588	0.09
2	51.00	88790	3.09	5	54.00	8666	0.30	8	57.05	4124	0.14
3	52.00	37879	1.32	6	55.00	20934	0.73	9	58.00	70786	2.46

1 / 3

#	m/z	Abs. In	Rel. Int.	#	m/z	Abs. In	Rel. Int.	#	m/z	Abs. In	Rel. Int.
10	59.00	16799	0.58	79	131.10	6262	0.22	148	200.05	12724	0.44
11	60.05	2242	0.08	80	132.10	6415	0.22	149	201.00	59105	2.05
12	61.05	8175	0.28	81	133.10	5788	0.20	150	202.00	20145	0.70
13	62.00	36782	1.28	82	134.10	13646	0.47	151	203.00	10553	0.37
14	63.00	81118	2.82	83	135.10	2930	0.10	152	204.00	10769	0.37
15	64.05	47440	1.65	84	136.10	1647	0.06	153	205.00	5138	0.18
16	65.00	347354	12.07	85	137.10	2422	0.08	154	206.00	4962	0.17
17	66.00	90618	3.15	86	138.10	3236	0.11	155	207.00	4775	0.17
18	67.00	60734	2.11	87	139.10	10484	0.36	156	208.00	4790	0.17
19	68.00	8705	0.30	88	140.05	22398	0.78	157	209.05	12641	0.44
20	69.00	7254	0.25	89	141.05	11555	0.40	158	210.00	37836	1.32
21	70.05	1908	0.07	90	142.10	8710	0.30	159	211.00	24878	0.86
22	71.00	1799	0.06	91	143.05	7207	0.25	160	212.00	22228	0.77
23	73.05	2138	0.07	92	144.05	6078	0.21	161	213.00	13057	0.45
24	74.00	13126	0.46	93	145.10	5254	0.18	162	214.00	5169	0.18
25	75.00	31081	1.08	94	146.05	8226	0.29	163	215.05	4160	0.14
26	76.00	65564	2.28	95	147.10	2791	0.10	164	216.00	4857	0.17
27	77.05	99537	3.46	96	148.05	10423	0.36	165	217.00	5455	0.19
28	78.05	38147	1.33	97	149.05	4223	0.15	166	218.00	3694	0.13
29	79.05	71165	2.47	98	150.05	7238	0.25	167	219.00	2378	0.08
30	80.05	9247	0.32	99	151.05	2863	0.10	168	220.00	4142	0.14
31	81.00	2569	0.09	100	152.05	7358	0.26	169	221.05	4865	0.17
32	82.05	1019	0.04	101	153.05	12486	0.43	170	222.00	31751	1.10
33	83.00	3257	0.11	102	154.05	10959	0.38	171	223.00	34863	1.21
34	84.05	2572	0.09	103	155.05	11797	0.41	172	224.05	20109	0.70
35	85.00	14740	0.51	104	156.05	7560	0.26	173	225.00	29622	1.03
36	86.05	7773	0.27	105	157.05	6711	0.23	174	226.05	18929	0.66
37	87.00	15089	0.52	106	158.00	6921	0.24	175	227.00	137474	4.78
38	88.05	15599	0.54	107	159.05	4619	0.16	176	228.00	25255	0.88
39	89.05	53190	1.85	108	160.00	7758	0.27	177	229.00	24881	0.86
40	90.15	112357	3.91	109	161.00	6388	0.22	178	230.00	5002	0.17
41	91.05	287693	100.00	110	162.00	4500	0.16	179	231.00	2238	0.08
42	92.05	230636	8.02	111	163.05	3148	0.11	180	232.05	1178	0.04
43	93.05	40423	1.41	112	164.05	15422	0.54	181	233.05	1399	0.05
44	94.05	7636	0.27	113	165.00	17342	0.60	182	234.00	4242	0.15
45	95.05	3967	0.14	114	166.00	10852	0.38	183	235.05	2892	0.10
46	97.05	1548	0.05	115	167.00	10249	0.36	184	236.05	4328	0.15
47	98.10	1519	0.05	116	168.00	10831	0.38	185	237.05	10533	0.37
48	99.05	5769	0.20	117	169.00	9295	0.32	186	238.05	22788	0.79
49	100.05	8225	0.29	118	170.00	9367	0.33	187	239.05	25471	0.89
50	101.05	14189	0.49	119	171.00	8498	0.30	188	240.05	21438	0.75
51	102.05	14438	0.50	120	172.00	5687	0.20	189	241.05	11551	0.40
52	103.10	9836	0.34	121	173.00	9319	0.32	190	242.05	5510	0.19
53	104.10	9887	0.34	122	174.05	6148	0.21	191	243.05	18237	0.63
54	105.10	22404	0.78	123	175.00	4734	0.16	192	244.05	22119	0.77
55	106.10	27550	0.96	124	176.00	3281	0.11	193	245.00	12543	0.44
56	107.10	74871	2.60	125	177.00	3540	0.12	194	246.05	2874	0.10
57	108.10	19250	0.67	126	178.00	6161	0.21	195	247.05	2007	0.07
58	109.05	2383	0.08	127	179.00	7633	0.27	196	248.05	3197	0.11
59	111.05	1120	0.04	128	180.00	8591	0.30	197	249.05	1998	0.07
60	112.10	2828	0.10	129	181.05	14527	0.50	198	250.10	2647	0.09
61	113.05	12115	0.42	130	182.00	11526	0.40	199	251.15	8215	0.29
62	114.05	12893	0.45	131	183.00	11242	0.39	200	252.05	52577	1.83
63	115.10	14262	0.50	132	184.00	9330	0.32	201	253.05	33318	1.16
64	116.10	9462	0.33	133	185.00	10642	0.37	202	254.05	19279	0.67
65	117.05	9417	0.33	134	186.00	14289	0.50	203	255.05	17162	0.60
66	118.10	5961	0.21	135	187.00	6441	0.22	204	256.05	6925	0.24
67	119.05	11809	0.41	136	188.00	7345	0.26	205	257.05	2839	0.10
68	120.05	13754	0.48	137	189.00	4554	0.16	206	258.05	1664	0.06
69	121.10	8554	0.30	138	190.00	2655	0.09	207	259.10	1710	0.06
70	122.05	3061	0.11	139	191.00	1910	0.07	208	260.10	1878	0.07
71	123.05	1181	0.04	140	192.00	5542	0.19	209	261.05	1698	0.06
72	124.15	1123	0.04	141	193.05	5085	0.18	210	262.05	1403	0.05
73	125.15	3772	0.13	142	194.00	18534	0.64	211	263.10	3169	0.11
74	126.10	13775	0.48	143	195.00	16514	0.57	212	264.05	1663	0.06
75	127.10	15196	0.53	144	196.00	15449	0.54	213	265.05	44634	1.55
76	128.05	12890	0.45	145	197.00	17388	0.60	214	266.05	28014	0.97
77	129.10	8782	0.31	146	198.05	15771	0.55	215	267.15	15280	0.53
78	130.10	9097	0.32	147	199.00	48042	1.67	216	268.05	113410	3.94

#	m/z	Abs. In	Rel. Int.
217	269.05	46480	1.62
218	270.05	11435	0.40
219	271.00	9975	0.35
220	272.05	4054	0.14
221	273.05	2987	0.10
222	274.05	2539	0.09
223	275.10	5218	0.18
224	276.05	7554	0.26
225	277.05	2032	0.07
226	278.05	1186	0.04
227	280.05	2198	0.08
228	281.05	1738	0.06
229	282.05	3002	0.10
230	283.05	36659	1.27
231	284.05	8954	0.31
232	285.05	4221	0.15
233	286.05	5109	0.18
234	287.05	3839	0.13
235	288.05	3628	0.13
236	289.10	3233	0.11
237	290.15	3492	0.12
238	291.10	33364	1.16
239	292.10	7265	0.25
240	293.10	1158	0.04
241	296.00	1705	0.06

#	m/z	Abs. In	Rel. Int.
242	297.05	2257	0.08
243	298.05	1962	0.07
244	299.05	3682	0.13
245	300.05	3583	0.12
246	301.05	3522	0.12
247	302.05	4851	0.17
248	303.05	2107	0.07
249	309.10	21812	0.76
250	310.05	5457	0.19
251	311.05	1439	0.05
252	312.05	1309	0.05
253	313.10	1430	0.05
254	314.05	2785	0.10
255	315.10	4049	0.14
256	316.15	2257	0.08
257	317.05	10417	0.36
258	318.05	3350	0.12
259	328.05	1798	0.06
260	329.05	1409	0.05
261	330.05	7014	0.24
262	331.05	2520	0.09
263	332.05	1517	0.05
264	333.10	1196	0.04
265	334.15	2454	0.09
266	335.05	10236	0.36

#	m/z	Abs. In	Rel. Int.
267	336.05	2260	0.08
268	342.10	3346	0.12
269	343.05	5674	0.20
270	344.05	3603	0.13
271	345.05	1551	0.05
272	357.05	1830	0.06
273	358.05	1072	0.04
274	359.15	1333	0.05
275	360.10	30857	1.07
276	361.05	8081	0.28
277	362.10	2148	0.07
278	374.05	7284	0.25
279	375.05	2454	0.09
280	376.05	1997	0.07
281	387.00	2233	0.08
282	388.05	1036	0.04
283	403.15	1950	0.07
284	404.05	42596	1.48
285	405.05	10889	0.38
286	406.10	1762	0.06
287	420.05	3029	0.11
288	421.15	1043	0.04
289	422.10	7140	0.25
290	423.05	1929	0.07

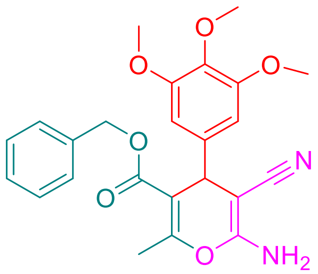

Compound 4d

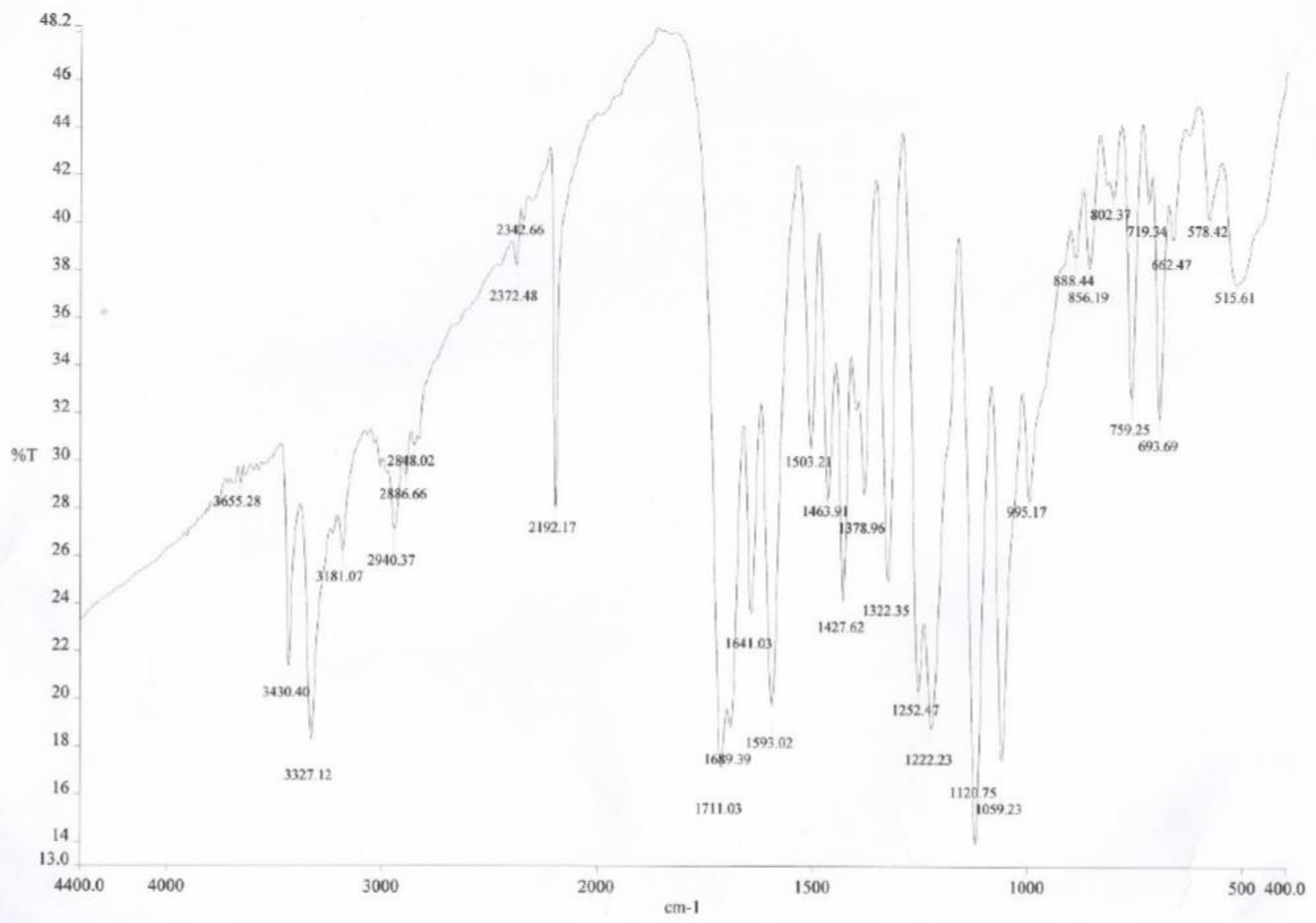

%T
cm-1
3655.28
3430.40
3327.12
3181.07
2940.37
2886.66
2848.02
2372.48
2342.66
2192.17
1711.03
1689.39
1641.03
1593.02
1503.21
1463.91
1427.62
1378.96
1322.35
1252.47
1222.23
1120.75
1059.23
995.17
888.44
856.19
802.37
759.25
719.34
693.69
662.47
578.42
515.61

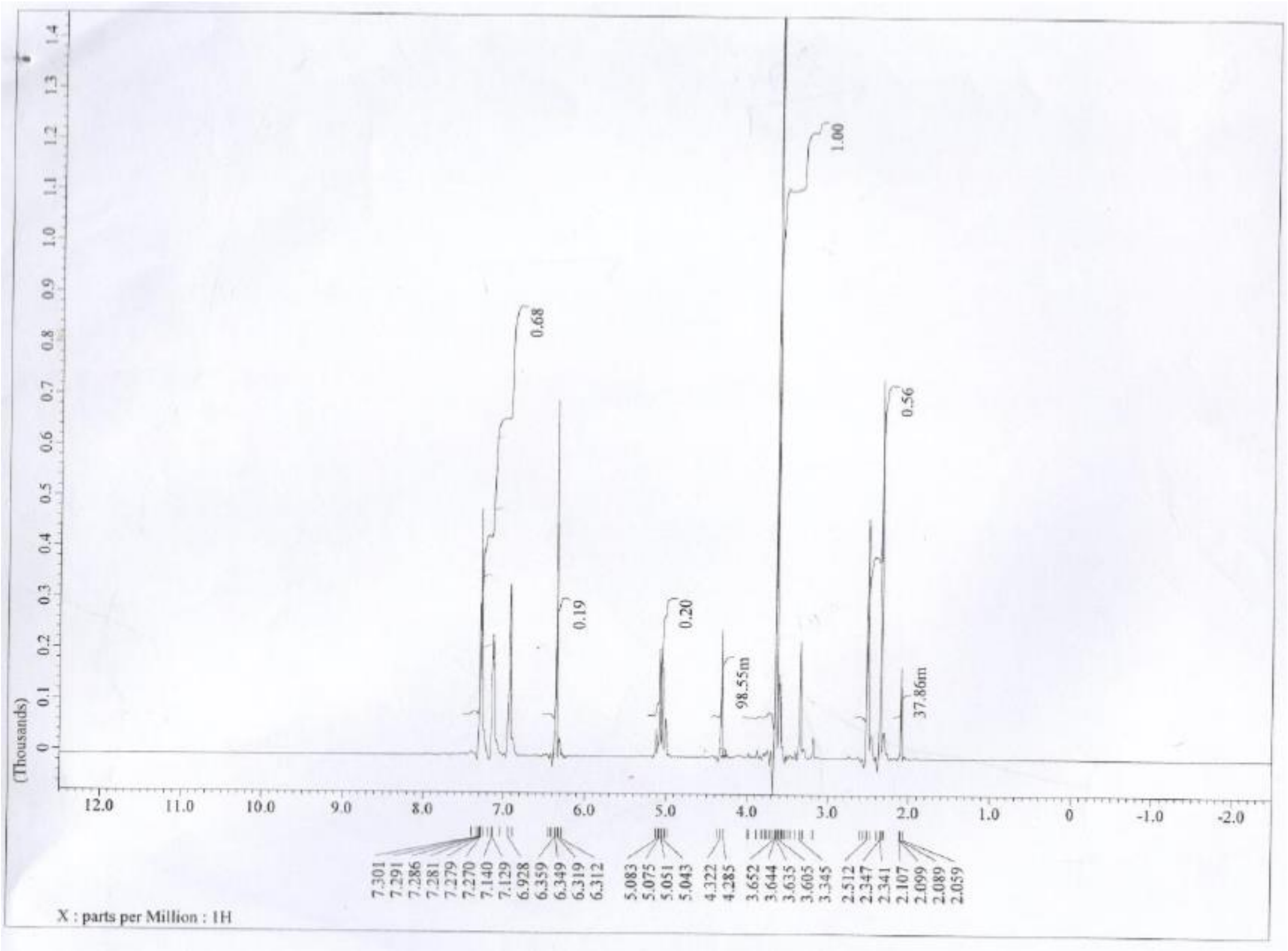
(Thousands)
0.68
0.19
0.20
98.55m
1.00
0.56
37.86m
7.301
7.291
7.286
7.281
7.279
7.270
7.140
7.129
6.928
6.359
6.349
6.319
6.312
5.083
5.075
5.051
5.043
4.322
4.285
3.652
3.644
3.635
3.605
3.345
2.512
2.347
2.341
2.107
2.099
2.089
2.059
X : parts per Million : 1H

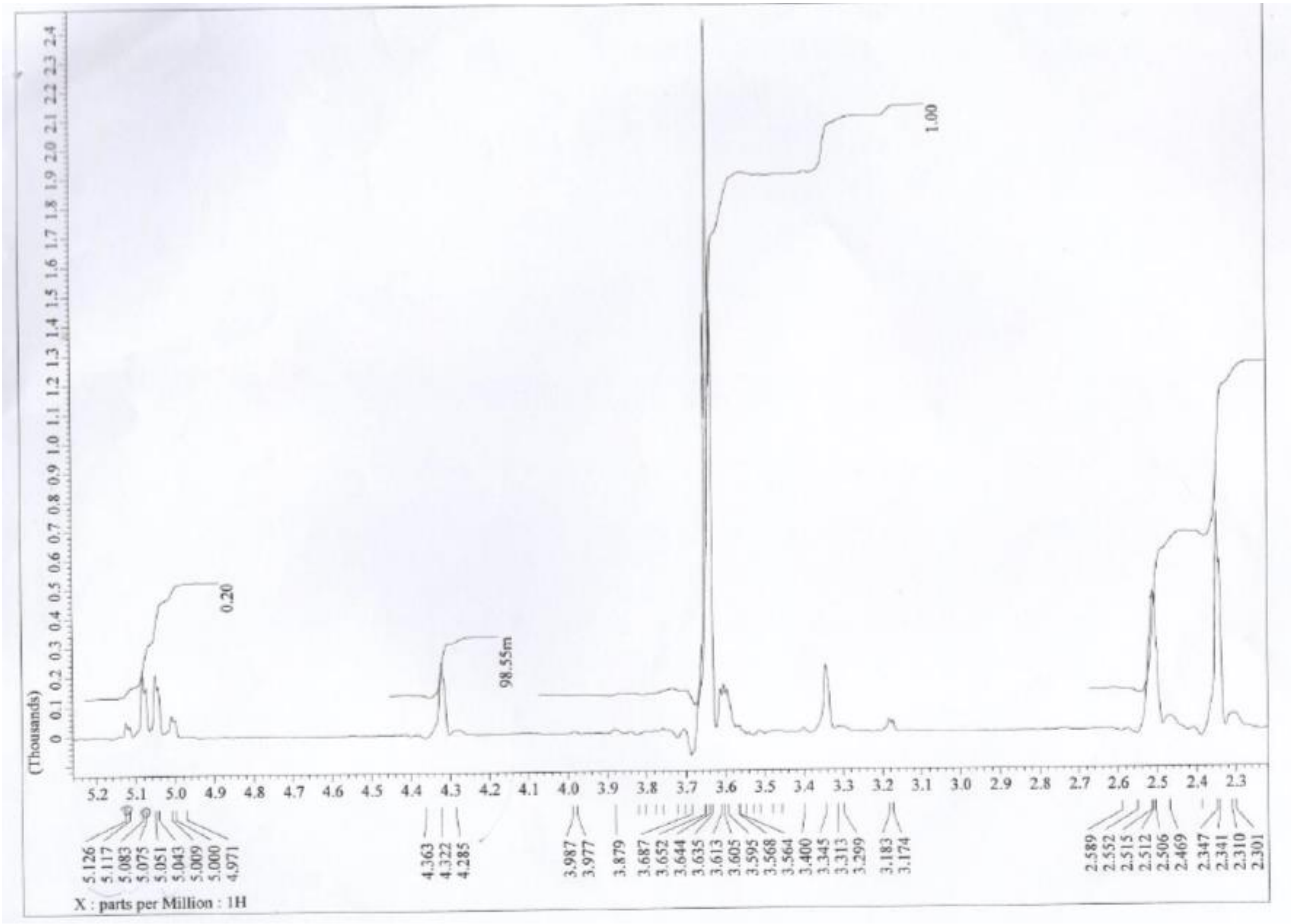
(Thousands)
X : parts per Million : 1H
0.20
98.55m
1.00
5.126
5.117
5.083
5.075
5.051
5.043
5.009
5.000
4.971
4.363
4.322
4.285
3.987
3.977
3.879
3.687
3.652
3.644
3.635
3.613
3.605
3.595
3.568
3.564
3.400
3.345
3.313
3.299
3.183
3.174
2.589
2.552
2.515
2.512
2.506
2.469
2.347
2.341
2.310
2.301

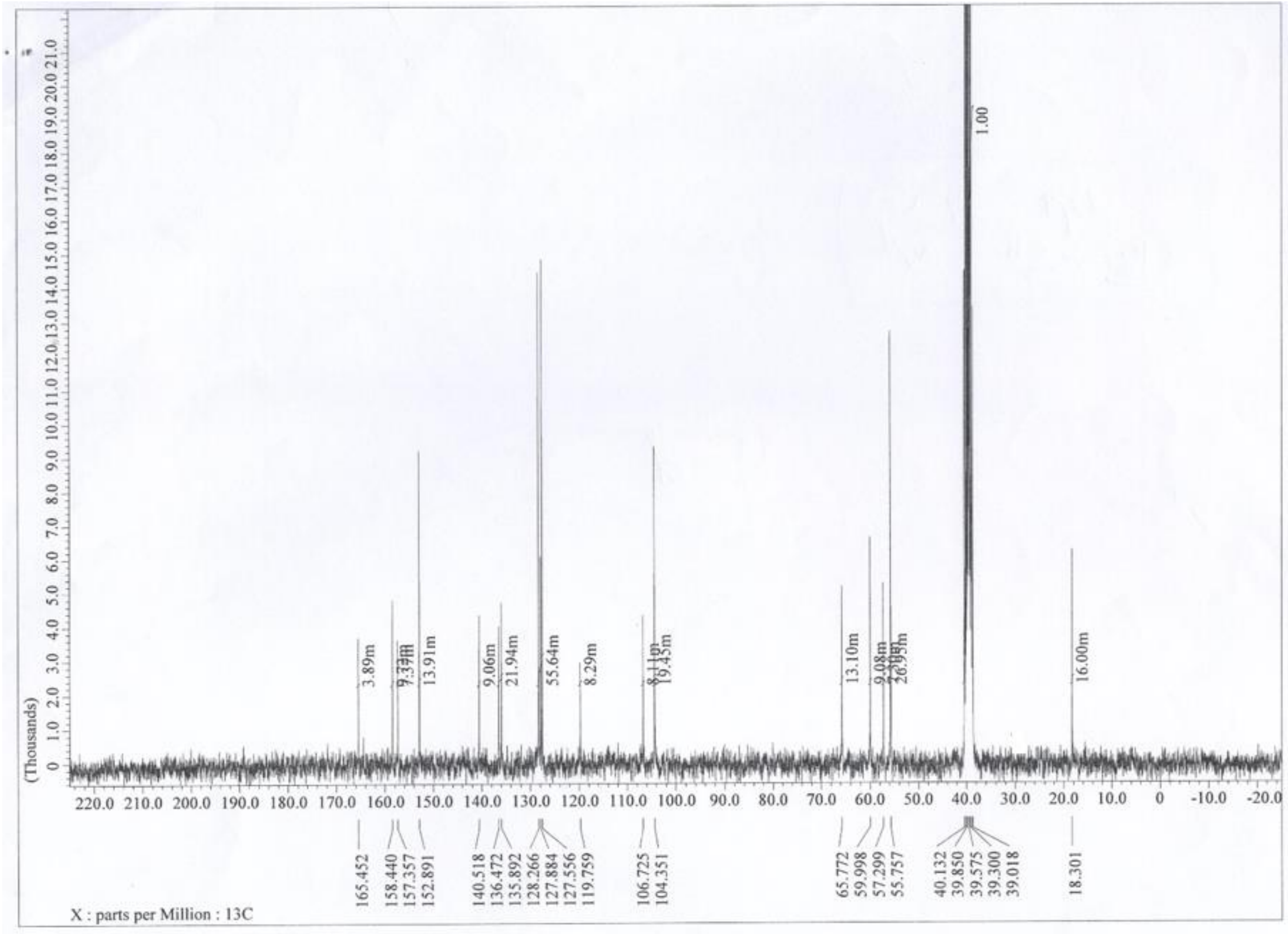
(Thousands)
X : parts per Million : 13C
165.452
158.440
157.357
152.891
140.518
136.472
135.892
128.266
127.884
127.556
119.759
106.725
104.351
65.772
59.998
57.299
55.757
40.132
39.850
39.575
39.300
39.018
18.301
3.89m
13.91m
9.06m
21.94m
55.64m
8.29m
19.45m
13.10m
9.08m
26.95m
16.00m
1.00

Line#:1 R.Time:3.5(Scan#:421)
MassPeaks:284
RawMode:Single 3.5(421) BasePeak:91(1421659)
BG Mode:None Group 1 - Event 1

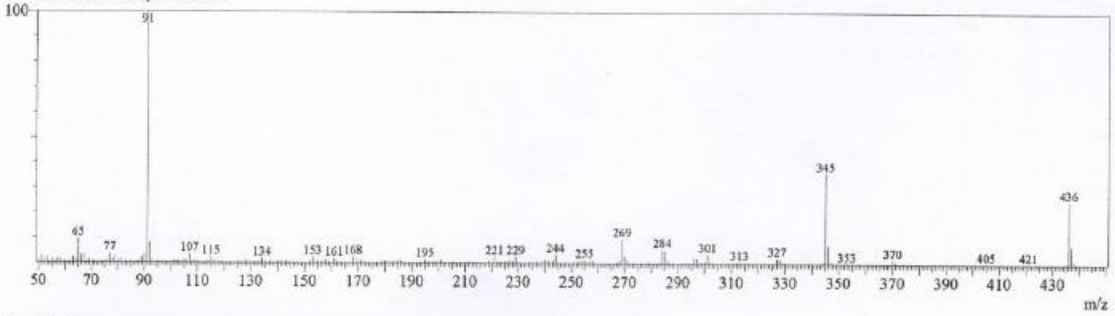

Mass Table
Line#:1 R.Time:3.5(Scan#:421)
MassPeaks:284
RawMode:Single 3.5(421) BasePeak:91(1421659)
BG Mode:None Group 1 - Event 1

#	m/z	Abs. In	Rel. Int.	#	m/z	Abs. In	Rel. Int.	#	m/z	Abs. In	Rel. Int.
1	50.00	16417	1.15	4	53.00	30078	2.12	7	56.05	5658	0.40
2	51.00	34822	2.45	5	54.05	5384	0.38	8	57.05	20646	1.45
3	52.05	14154	1.00	6	55.00	20806	1.46	9	58.00	19583	1.38

1 / 3

#	m/z	Abs. In	Rel. Int.
10	59.00	9838	0.69
11	60.00	5751	0.40
12	61.00	3999	0.28
13	62.05	7849	0.55
14	63.00	30351	2.13
15	64.05	19015	1.34
16	65.00	132002	9.29
17	66.00	43871	3.09
18	67.00	46948	3.30
19	68.00	6567	0.46
20	69.00	17145	1.21
21	70.10	3879	0.27
22	71.05	9579	0.67
23	72.05	1386	0.10
24	73.00	5844	0.41
25	74.05	4466	0.31
26	75.00	11596	0.82
27	76.05	14067	0.99
28	77.00	49249	3.46
29	78.05	17589	1.24
30	79.00	35305	2.48
31	80.05	5666	0.40
32	81.05	20700	1.46
33	82.05	4578	0.32
34	83.05	7561	0.53
35	84.05	4506	0.32
36	85.05	11348	0.80
37	86.00	2946	0.21
38	87.00	5764	0.41
39	88.00	10929	0.77
40	89.00	28300	1.99
41	90.05	42923	3.02
42	91.05	142165	100.00
43	92.05	115599	8.13
44	93.00	15351	1.08
45	94.05	4861	0.34
46	95.05	10198	0.72
47	96.10	5086	0.36
48	97.05	7006	0.49
49	98.05	4183	0.29
50	99.05	5111	0.36
51	100.00	7138	0.50
52	101.00	12394	0.87
53	102.05	12492	0.88
54	103.05	11579	0.81
55	104.10	6551	0.46
56	105.05	17530	1.23
57	106.05	10605	0.75
58	107.05	47321	3.33
59	108.05	11821	0.83
60	109.05	15308	1.08
61	110.05	7499	0.53
62	111.10	4602	0.32
63	112.10	2841	0.20
64	113.05	6273	0.44
65	114.05	9747	0.69
66	115.05	30877	2.17
67	116.05	13010	0.92
68	117.05	10187	0.72
69	118.05	5813	0.41
70	119.05	7000	0.49
71	120.05	3300	0.23
72	121.05	5157	0.36
73	122.05	4383	0.31
74	123.05	3499	0.25
75	124.05	2943	0.21
76	125.05	8581	0.60
77	126.05	5127	0.36
78	127.05	11877	0.84

#	m/z	Abs. In	Rel. Int.
79	128.05	12791	0.90
80	129.05	10260	0.72
81	130.05	7826	0.55
82	131.05	6852	0.48
83	132.05	4511	0.32
84	133.05	10013	0.70
85	134.05	22366	1.57
86	135.05	6895	0.48
87	136.05	1930	0.14
88	137.05	7909	0.56
89	138.05	3375	0.24
90	139.10	5937	0.42
91	140.05	9834	0.69
92	141.05	9763	0.69
93	142.05	6782	0.48
94	143.05	9266	0.65
95	144.05	7705	0.54
96	145.05	6114	0.43
97	146.05	4383	0.31
98	147.05	4356	0.31
99	148.05	2731	0.19
100	149.05	4816	0.34
101	150.05	2147	0.15
102	151.05	3737	0.26
103	152.05	7186	0.51
104	153.00	29868	2.10
105	154.00	10734	0.76
106	155.00	9927	0.70
107	156.00	8934	0.63
108	157.00	8858	0.62
109	158.00	17556	1.23
110	159.00	6710	0.47
111	160.05	3789	0.27
112	161.00	23513	1.65
113	162.00	6953	0.49
114	163.00	6225	0.44
115	164.05	6294	0.44
116	165.00	4503	0.32
117	166.00	3268	0.23
118	167.05	5055	0.36
119	168.00	32431	2.28
120	169.00	17681	1.24
121	170.00	8738	0.61
122	171.00	14490	1.02
123	172.00	9857	0.69
124	172.95	6898	0.49
125	174.00	4505	0.32
126	175.00	3559	0.25
127	176.00	2938	0.21
128	176.95	4984	0.35
129	178.00	6518	0.46
130	178.95	11311	0.80
131	180.00	11470	0.81
132	181.00	8881	0.62
133	182.00	9361	0.66
134	183.00	8583	0.60
135	184.00	8900	0.63
136	185.00	14049	0.99
137	185.95	13281	0.93
138	186.95	5783	0.41
139	187.95	4408	0.31
140	189.00	3599	0.25
141	190.00	3476	0.24
142	190.95	3311	0.23
143	192.00	3118	0.22
144	193.00	4743	0.33
145	194.00	4239	0.30
146	194.95	19524	1.37
147	196.00	8566	0.60

#	m/z	Abs. In	Rel. Int.
148	197.00	7390	0.52
149	198.00	6809	0.48
150	199.00	8866	0.62
151	200.00	9071	0.64
152	200.95	18001	1.27
153	202.00	5535	0.39
154	202.95	3402	0.24
155	204.00	3350	0.24
156	205.00	7159	0.50
157	205.95	8957	0.63
158	207.00	3775	0.27
159	208.00	6223	0.44
160	209.00	5322	0.37
161	210.00	7070	0.50
162	210.95	9656	0.68
163	212.00	8545	0.60
164	213.00	7340	0.52
165	214.00	6862	0.48
166	215.00	6857	0.48
167	216.00	4178	0.29
168	216.95	3626	0.26
169	218.00	3226	0.23
170	219.00	13375	0.94
171	220.05	3648	0.26
172	221.00	33553	2.36
173	222.00	8522	0.60
174	223.00	9566	0.67
175	224.00	7849	0.55
176	225.00	11353	0.80
177	225.95	12189	0.86
178	227.00	20292	1.43
179	228.00	10273	0.72
180	229.00	34004	2.39
181	229.95	8142	0.57
182	231.00	4401	0.31
183	232.00	2473	0.17
184	233.00	4874	0.34
185	234.00	7090	0.50
186	235.00	6638	0.47
187	236.00	8090	0.57
188	237.00	9415	0.66
189	238.00	7449	0.52
190	239.00	12734	0.90
191	240.00	17095	1.20
192	241.00	15052	1.06
193	242.00	11953	0.84
194	243.05	14401	1.01
195	244.00	47415	3.34
196	245.00	10012	0.70
197	246.00	3196	0.22
198	247.00	4297	0.30
199	248.00	6086	0.43
200	249.00	3834	0.27
201	250.05	1764	0.12
202	251.00	12260	0.86
203	252.00	8940	0.63
204	253.00	15105	1.06
205	254.00	14454	1.02
206	255.00	17394	1.22
207	256.00	11180	0.79
208	257.05	15801	1.11
209	258.05	13862	0.98
210	259.00	6529	0.46
211	260.05	2898	0.20
212	261.00	3342	0.24
213	262.05	2487	0.17
214	263.00	4575	0.32
215	264.00	2044	0.14
216	265.05	2354	0.17

#	m/z	Abs. In	Rel. Int.	#	m/z	Abs. In	Rel. Int.	#	m/z	Abs. In	Rel. Int.
217	266.00	3460	0.24	240	295.05	3289	0.23	263	330.00	5634	0.40
218	267.00	8970	0.63	241	296.00	32490	2.29	264	331.00	1713	0.12
219	268.05	16966	1.19	242	297.00	30666	2.16	265	339.00	4470	0.31
220	269.05	137905	9.70	243	297.95	8769	0.62	266	340.00	1302	0.09
221	270.00	42678	3.00	244	299.00	8458	0.59	267	344.05	9200	0.65
222	271.00	18372	1.29	245	300.05	14387	1.01	268	345.00	514161	36.17
223	272.00	7065	0.50	246	301.05	50508	3.55	269	346.00	102474	7.21
224	273.05	2592	0.18	247	302.00	15559	1.09	270	347.00	16686	1.17
225	274.00	1354	0.10	248	303.00	4839	0.34	271	348.00	2228	0.16
226	275.05	1805	0.13	249	304.00	1046	0.07	272	353.05	1021	0.07
227	276.05	1501	0.11	250	309.00	2014	0.14	273	369.05	1405	0.10
228	278.05	1275	0.09	251	310.00	2156	0.15	274	370.00	24138	1.70
229	279.00	10719	0.75	252	311.00	5385	0.38	275	371.00	6089	0.43
230	280.00	2969	0.21	253	312.00	6778	0.48	276	372.00	1364	0.10
231	281.00	5566	0.39	254	313.00	8745	0.62	277	393.00	1713	0.12
232	282.00	4290	0.30	255	314.00	3364	0.24	278	405.00	3318	0.23
233	283.05	8215	0.58	256	315.00	3092	0.22	279	421.00	1383	0.10
234	284.00	78078	5.49	257	316.00	1194	0.08	280	435.05	8309	0.58
235	285.00	69441	4.88	258	317.00	1308	0.09	281	436.05	353768	24.88
236	286.00	17247	1.21	259	326.05	1388	0.10	282	437.05	99249	6.98
237	287.00	7780	0.55	260	327.00	29538	2.08	283	438.05	17851	1.26
238	288.00	2799	0.20	261	328.00	27198	1.91	284	439.05	2519	0.18
239	293.95	3144	0.22	262	329.00	12684	0.89				

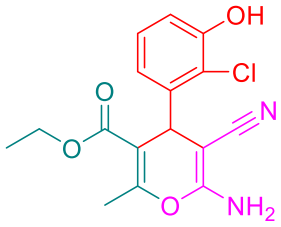

Compound 4g

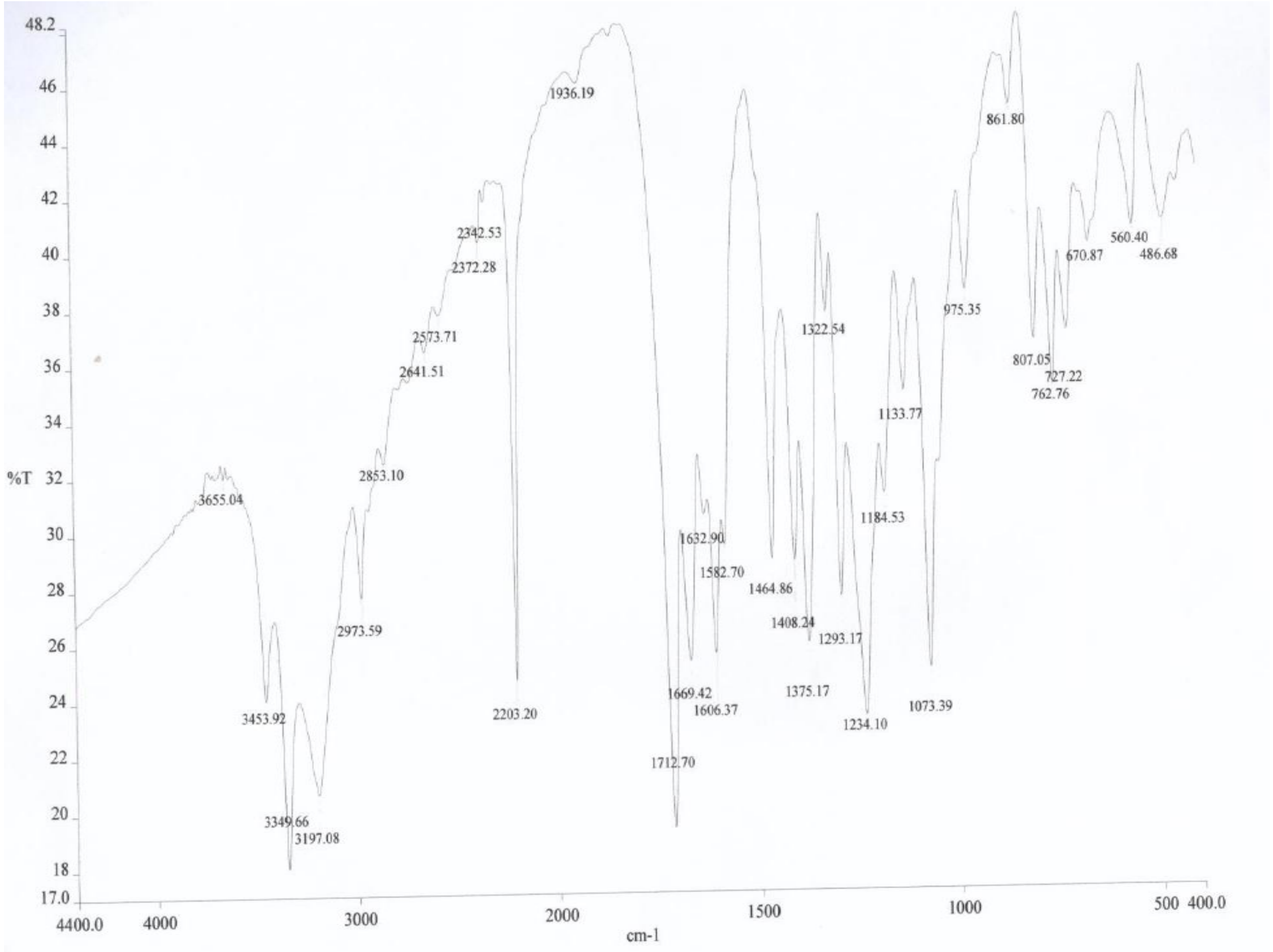
%T
cm-1
48.2
46
44
42
40
38
36
34
32
30
28
26
24
22
20
18
17.0
4400.0
4000
3000
2000
1500
1000
500
400.0
3655.04
3453.92
3349.66
3197.08
2973.59
2853.10
2641.51
2573.71
2372.28
2342.53
2203.20
1936.19
1712.70
1669.42
1632.90
1606.37
1582.70
1464.86
1408.24
1375.17
1322.54
1293.17
1234.10
1184.53
1133.77
1073.39
975.35
861.80
807.05
762.76
727.22
670.87
560.40
486.68

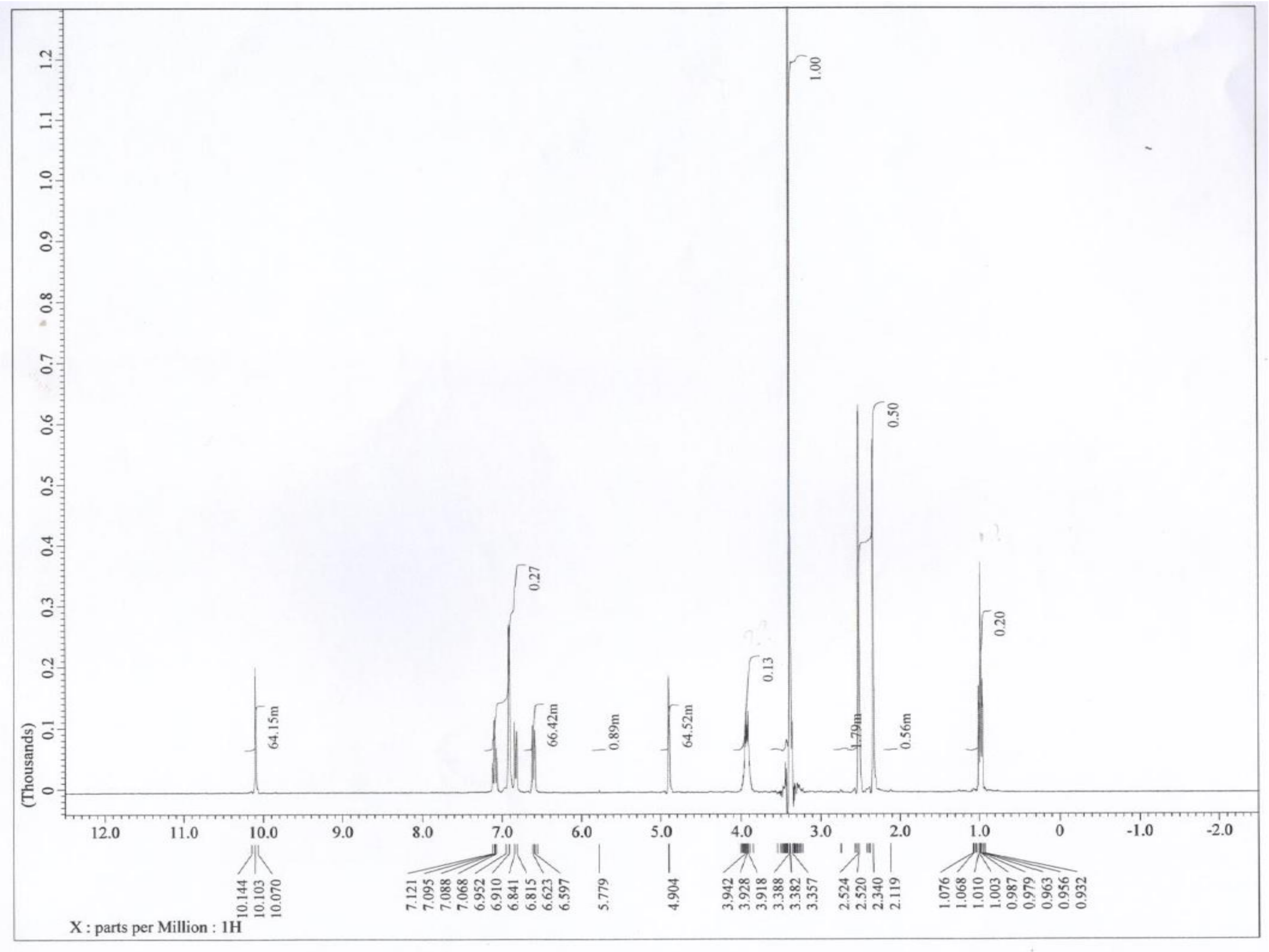
(Thousands)
X : parts per Million : 1H
1.00
0.50
0.27
0.20
0.13
64.15m
66.42m
0.89m
64.52m
1.79m
0.56m
10.144
10.103
10.070
7.121
7.095
7.088
7.068
6.952
6.910
6.841
6.815
6.623
6.597
5.779
4.904
3.942
3.928
3.918
3.388
3.382
3.357
2.524
2.520
2.340
2.119
1.076
1.068
1.010
1.003
0.987
0.979
0.963
0.956
0.932

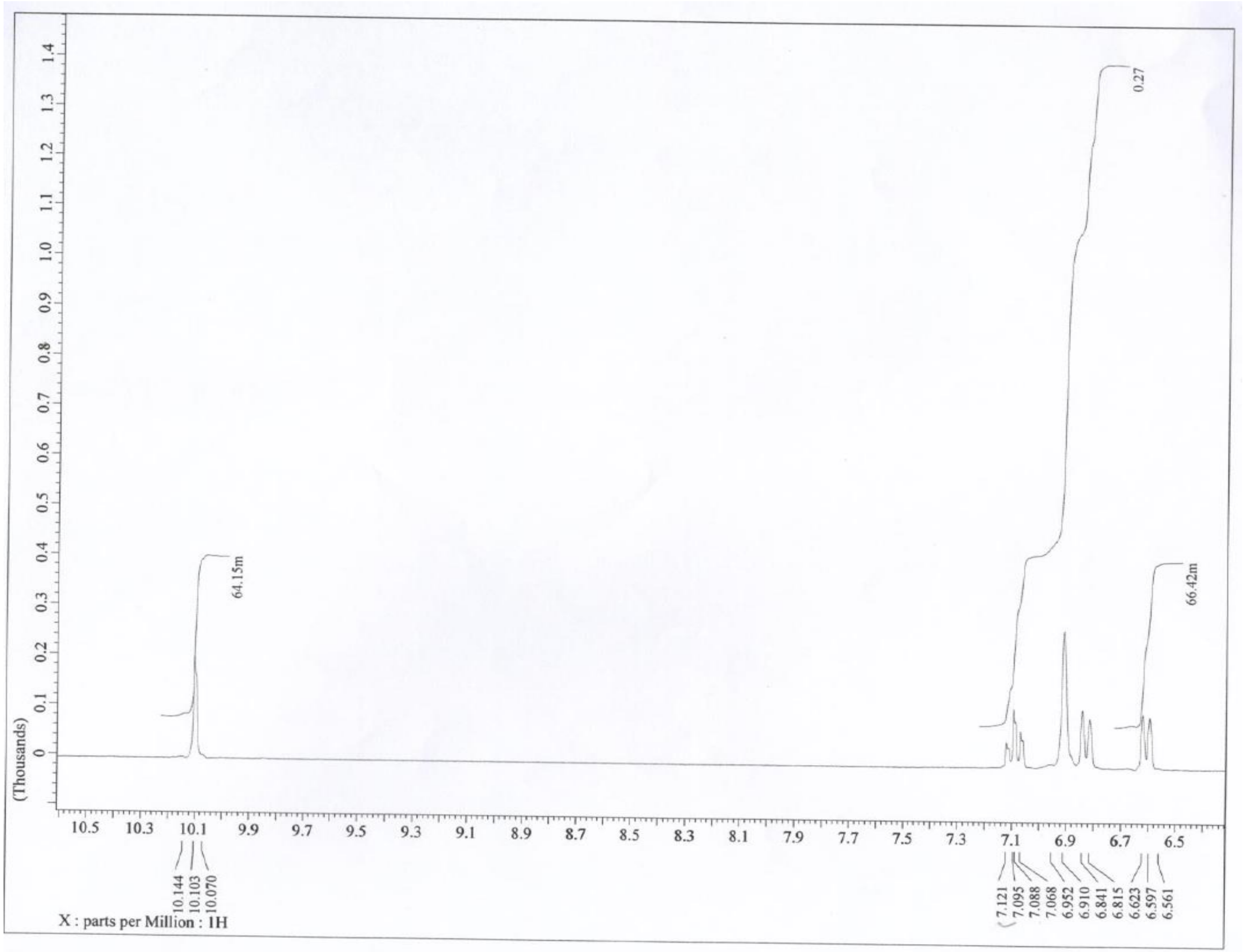
(Thousands)
64.15m
0.27
66.42m
10.144
10.103
10.070
7.121
7.095
7.088
7.068
6.952
6.910
6.841
6.815
6.623
6.597
6.561
X : parts per Million : 1H

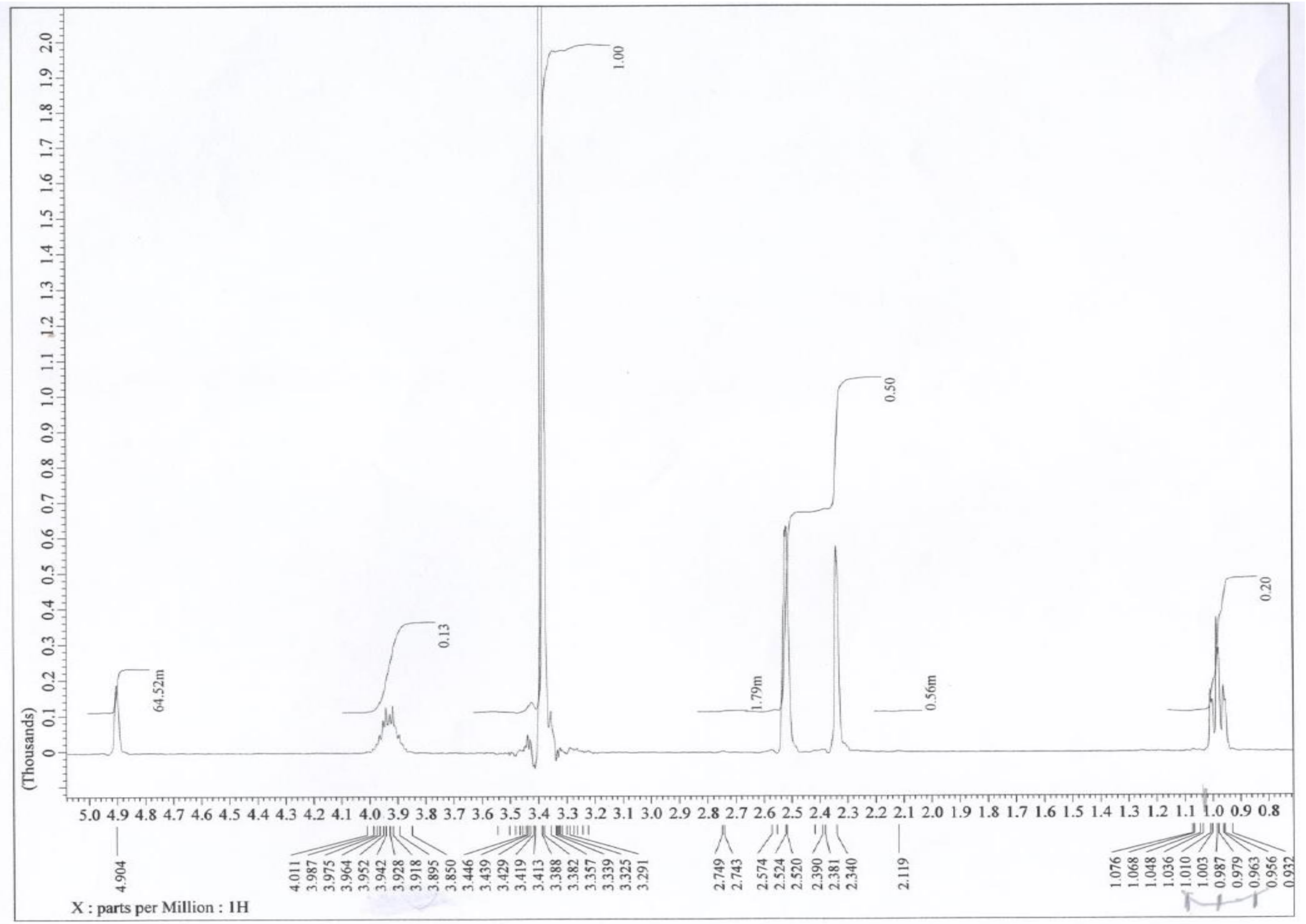
(Thousands)
X : parts per Million : 1H
64.52m
0.13
1.00
1.79m
0.50
0.56m
0.20
4.904
4.011 3.987 3.975 3.964 3.952 3.942 3.928 3.918 3.895 3.850
3.446 3.439 3.429 3.419 3.413 3.388 3.382 3.357 3.339 3.325 3.291
2.749 2.743
2.574 2.524 2.520
2.390 2.381 2.340
2.119
1.076 1.068 1.048 1.036 1.010 1.003 0.987 0.979 0.963 0.956 0.932

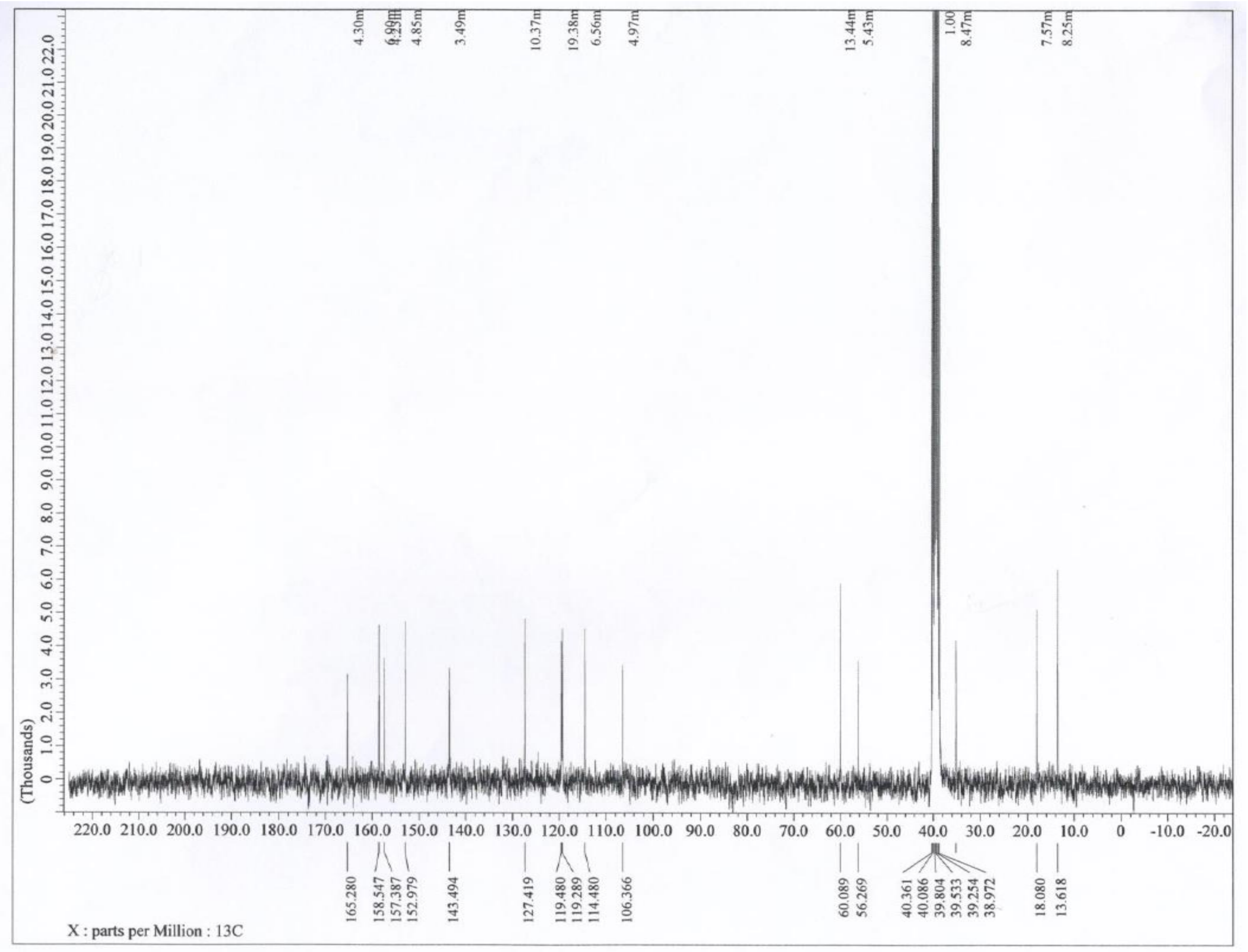
4.30m
6.90m
4.25m
4.85m
3.49m
10.37m
19.38m
6.56m
4.97m
13.44m
5.43m
1.00
8.47m
7.57m
8.25m
(Thousands)
0 1.0 2.0 3.0 4.0 5.0 6.0 7.0 8.0 9.0 10.0 11.0 12.0 13.0 14.0 15.0 16.0 17.0 18.0 19.0 20.0 21.0 22.0
220.0 210.0 200.0 190.0 180.0 170.0 160.0 150.0 140.0 130.0 120.0 110.0 100.0 90.0 80.0 70.0 60.0 50.0 40.0 30.0 20.0 10.0 0 -10.0 -20.0
165.280
158.547
157.387
152.979
143.494
127.419
119.480
119.289
114.480
106.366
60.089
56.269
40.361
40.086
39.804
39.533
39.254
38.972
18.080
13.618
X : parts per Million : 13C

Line#:1 R.Time:2.3(Scan#:272)
MassPeaks:260
RawMode:Single 2.3(272) BasePeak:207(829071)
BG Mode:None Group 1 - Event 1

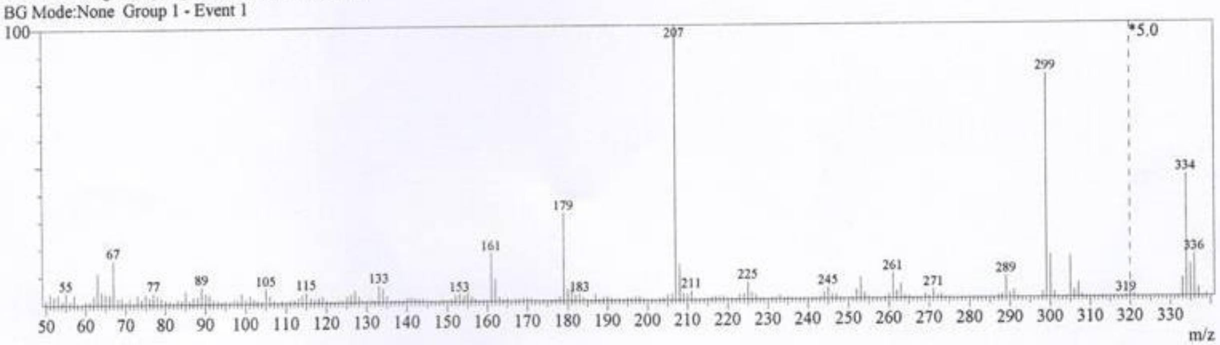

Mass Table
Line#:1 R.Time:2.3(Scan#:272)
MassPeaks:260
RawMode:Single 2.3(272) BasePeak:207(829071)
BG Mode:None Group 1 - Event 1

#	m/z	Abs. In	Rel. Int.	#	m/z	Abs. In	Rel. Int.	#	m/z	Abs. In	Rel. Int.
1	50.00	12580	1.52	4	53.00	29759	3.59	7	56.05	9031	1.09
2	51.00	33724	4.07	5	54.05	10162	1.23	8	57.05	29743	3.59
3	52.00	23404	2.82	6	55.00	34367	4.15	9	58.00	3162	0.38

1 / 3

#	m/z	Abs. In	Rel. Int.
10	59.05	2863	0.35
11	60.00	8386	1.01
12	61.00	8942	1.08
13	62.05	27374	3.30
14	63.00	94250	11.37
15	64.00	38694	4.67
16	65.00	31215	3.77
17	66.05	28561	3.44
18	67.00	131818	15.90
19	68.00	17562	2.12
20	69.05	20440	2.47
21	70.10	7735	0.93
22	71.05	17431	2.10
23	72.05	4835	0.58
24	73.00	27729	3.34
25	74.05	13503	1.63
26	75.00	30618	3.69
27	76.00	18294	2.21
28	77.05	32208	3.88
29	78.05	24872	3.00
30	79.05	16936	2.04
31	80.05	11704	1.41
32	81.05	9232	1.11
33	82.05	5326	0.64
34	83.05	12337	1.49
35	84.05	11628	1.40
36	85.05	40152	4.84
37	86.05	8478	1.02
38	87.00	19516	2.35
39	88.05	21425	2.58
40	89.05	50056	6.04
41	90.05	31307	3.78
42	91.00	25719	3.10
43	92.00	10838	1.31
44	93.05	10631	1.28
45	94.05	6146	0.74
46	95.05	8862	1.07
47	96.10	5182	0.63
48	97.05	13338	1.61
49	98.05	11249	1.36
50	99.00	30553	3.69
51	100.00	13305	1.60
52	101.05	22182	2.68
53	102.05	13774	1.66
54	103.05	8349	1.01
55	104.15	6637	0.80
56	105.05	44148	5.32
57	106.05	22447	2.71
58	107.10	8095	0.98
59	108.10	3945	0.48
60	109.05	6182	0.75
61	110.10	3652	0.44
62	111.10	8871	1.07
63	112.10	8394	1.01
64	113.10	14161	1.71
65	114.05	24038	2.90
66	115.05	31440	3.79
67	116.10	13782	1.66
68	117.05	10933	1.32
69	118.05	13935	1.68
70	119.05	18896	2.28
71	120.05	6139	0.74
72	121.05	3567	0.43
73	122.05	3226	0.39
74	123.05	8580	1.03
75	124.10	6342	0.76
76	125.05	16775	2.02
77	126.05	23831	2.87
78	127.05	36347	4.38

#	m/z	Abs. In	Rel. Int.
79	128.05	19073	2.30
80	129.10	8734	1.05
81	130.10	5212	0.63
82	131.10	8001	0.97
83	132.15	5269	0.64
84	133.05	49712	6.00
85	134.05	40595	4.90
86	135.05	17741	2.14
87	136.05	3767	0.45
88	137.10	6034	0.73
89	138.05	5305	0.64
90	139.10	5959	0.72
91	140.10	10678	1.29
92	141.05	12409	1.50
93	142.05	10379	1.25
94	143.05	9690	1.17
95	144.05	8765	1.06
96	145.00	4306	0.52
97	146.05	1908	0.23
98	147.05	2583	0.31
99	148.05	1754	0.21
100	149.05	6442	0.78
101	150.05	4402	0.53
102	151.05	6386	0.77
103	152.05	18824	2.27
104	153.00	21012	2.53
105	154.05	17632	2.13
106	155.00	24708	2.98
107	156.05	13714	1.65
108	157.00	7137	0.86
109	158.00	2940	0.35
110	159.05	4587	0.55
111	160.05	5888	0.71
112	161.00	143836	17.35
113	162.00	66228	7.99
114	163.00	13100	1.58
115	164.00	5783	0.70
116	165.00	7846	0.95
117	166.00	3385	0.41
118	167.00	4910	0.59
119	168.00	4959	0.60
120	169.00	9634	1.16
121	170.00	8362	1.01
122	171.00	7404	0.89
123	172.00	2743	0.33
124	173.00	1574	0.19
125	174.00	1649	0.20
126	175.00	1281	0.15
127	175.95	2446	0.30
128	177.00	5031	0.61
129	178.05	12054	1.45
130	179.00	264796	31.94
131	180.00	35361	4.27
132	180.95	27130	3.27
133	182.00	17897	2.16
134	183.00	18804	2.27
135	184.00	9423	1.14
136	185.05	4240	0.51
137	186.05	2261	0.27
138	187.00	18617	2.25
139	188.00	4212	0.51
140	189.00	7991	0.96
141	189.95	11751	1.42
142	190.95	7259	0.88
143	191.95	4851	0.59
144	192.95	5132	0.62
145	194.00	2793	0.34
146	195.00	7830	0.94
147	196.00	5244	0.63

#	m/z	Abs. In	Rel. Int.
148	197.00	11108	1.34
149	198.00	10428	1.26
150	199.00	5889	0.71
151	200.00	2922	0.35
152	201.00	1548	0.19
153	201.95	1380	0.17
154	202.95	2363	0.29
155	203.95	5686	0.69
156	205.00	15412	1.86
157	206.05	18398	2.22
158	207.00	829071	100.00
159	208.00	107722	12.99
160	209.00	20894	2.52
161	210.00	18066	2.18
162	211.00	26886	3.24
163	212.00	5946	0.72
164	213.05	2958	0.36
165	214.00	2623	0.32
166	215.00	2566	0.31
167	216.00	9060	1.09
168	216.95	9676	1.17
169	217.95	11269	1.36
170	218.95	10955	1.32
171	219.95	5979	0.72
172	220.95	4932	0.59
173	222.05	2509	0.30
174	222.95	14558	1.76
175	224.00	15655	1.89
176	225.00	52988	6.39
177	226.00	20084	2.42
178	227.00	12911	1.56
179	228.00	5697	0.69
180	229.05	3765	0.45
181	230.00	2223	0.27
182	231.00	4532	0.55
183	232.00	5716	0.69
184	233.00	12332	1.49
185	234.00	5188	0.63
186	235.00	6452	0.78
187	236.00	4726	0.57
188	237.00	4006	0.48
189	238.00	3158	0.38
190	239.10	3007	0.36
191	240.05	1077	0.13
192	241.05	3251	0.39
193	242.00	2473	0.30
194	243.05	4589	0.55
195	244.00	19236	2.32
196	244.95	33614	4.05
197	245.95	13294	1.60
198	246.95	13617	1.64
199	247.95	3886	0.47
200	249.00	1802	0.22
201	250.05	1224	0.15
202	251.05	3528	0.43
203	252.05	26603	3.21
204	253.05	64215	7.75
205	254.00	17753	2.14
206	255.05	6527	0.79
207	256.10	2382	0.29
208	257.15	1635	0.20
209	259.05	4490	0.54
210	260.05	12926	1.56
211	261.00	75047	9.05
212	262.00	21842	2.63
213	263.00	42406	5.11
214	264.00	8638	1.04
215	265.00	8282	1.00
216	266.05	1521	0.18

#	m/z	Abs. In	Rel. Int.
217	267.10	1950	0.24
218	268.05	1082	0.13
219	269.05	12588	1.52
220	270.00	6604	0.80
221	271.05	25605	3.09
222	272.05	7911	0.95
223	273.00	12353	1.49
224	274.00	2879	0.35
225	275.00	3996	0.48
226	276.00	1252	0.15
227	280.00	1262	0.15
228	281.05	1120	0.14
229	282.05	1216	0.15
230	285.10	1295	0.16
231	287.00	8543	1.03

#	m/z	Abs. In	Rel. Int.
232	288.05	9723	1.17
233	289.00	65234	7.87
234	290.00	13959	1.68
235	291.00	22985	2.77
236	292.00	4551	0.55
237	293.00	1378	0.17
238	295.00	1078	0.13
239	297.10	2825	0.34
240	298.15	18117	2.19
241	299.10	676655	81.62
242	300.05	127443	15.37
243	301.05	17116	2.06
244	302.05	2077	0.25
245	304.05	1926	0.23
246	305.00	123962	14.95

#	m/z	Abs. In	Rel. Int.
247	306.00	21266	2.57
248	307.00	42828	5.17
249	307.95	7615	0.92
250	309.00	1396	0.17
251	313.15	1467	0.18
252	319.00	3914	0.47
253	320.00	1220	0.15
254	321.00	1476	0.18
255	333.05	10997	1.33
256	334.00	74137	8.94
257	335.00	19362	2.34
258	336.00	25348	3.06
259	337.00	5242	0.63
260	338.95	1472	0.18

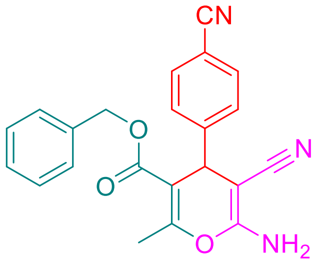

Compound 4k

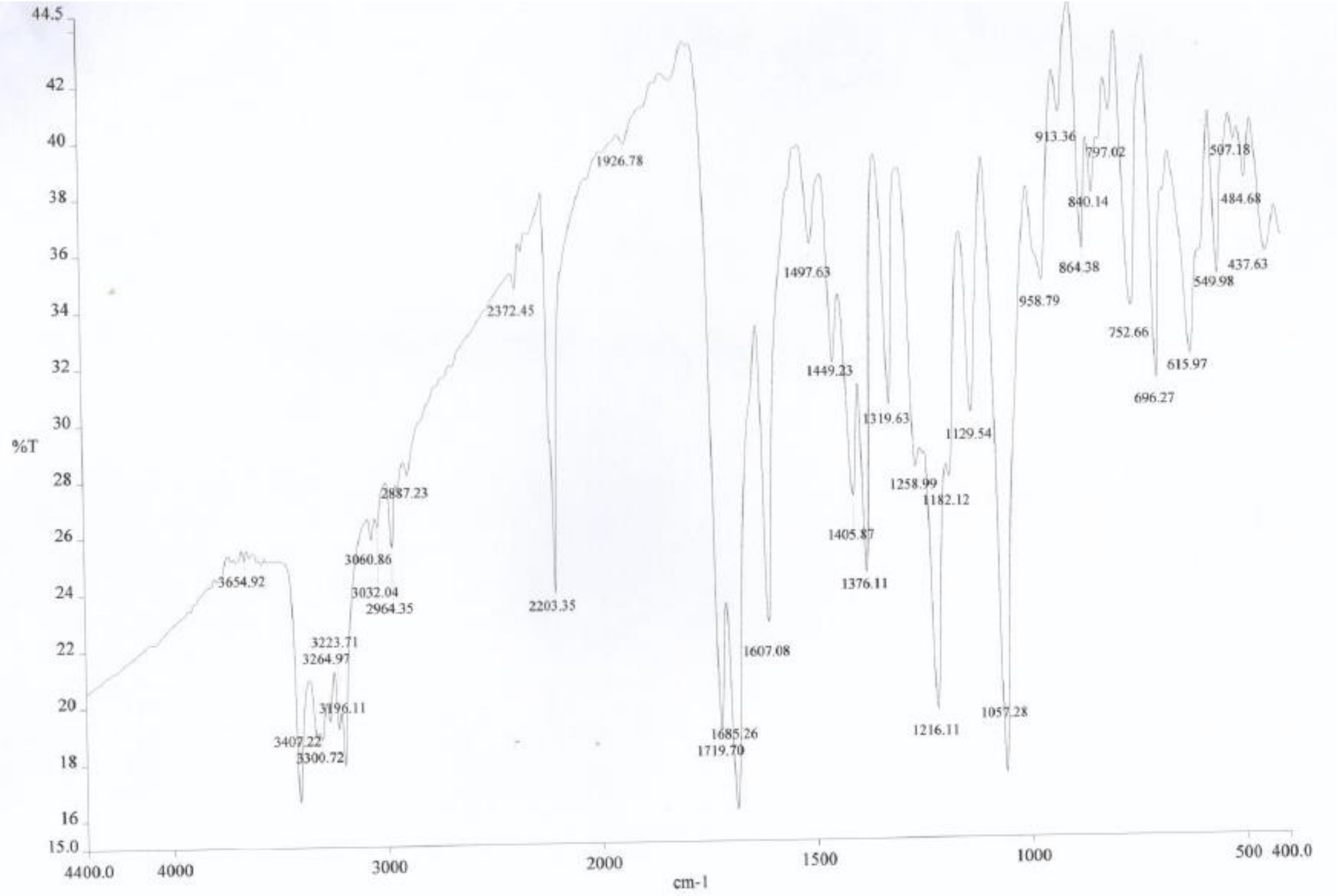

%T
cm-1
3654.92
3407.22
3300.72
3264.97
3223.71
3196.11
3060.86
3032.04
2964.35
2887.23
2372.45
2203.35
1926.78
1719.70
1685.26
1607.08
1497.63
1449.23
1405.87
1376.11
1319.63
1258.99
1216.11
1182.12
1129.54
1057.28
958.79
913.36
864.38
840.14
797.02
752.66
696.27
615.97
549.98
507.18
484.68
437.63

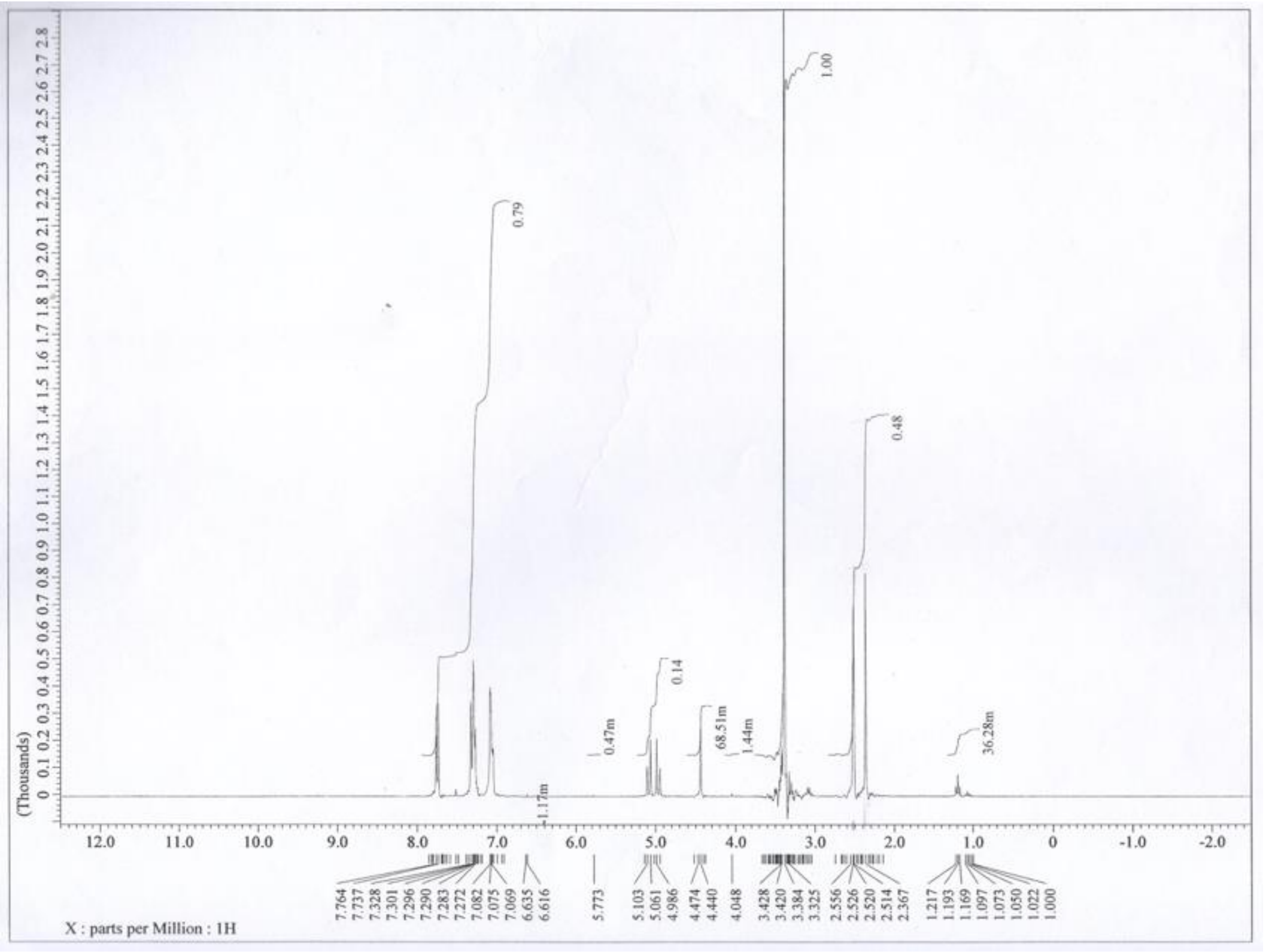
(Thousands)
X : parts per Million : 1H
0.79
1.00
0.48
0.14
0.47m
68.51m
1.44m
36.28m
1.17m
7.764 7.737 7.328 7.301 7.296 7.290 7.283 7.272 7.082 7.075 7.069 6.635 6.616
5.773
5.103 5.061 4.986
4.474 4.440
4.048
3.428 3.420 3.384 3.325
2.556 2.526 2.520 2.514 2.367
1.217 1.193 1.169 1.097 1.073 1.050 1.022 1.000

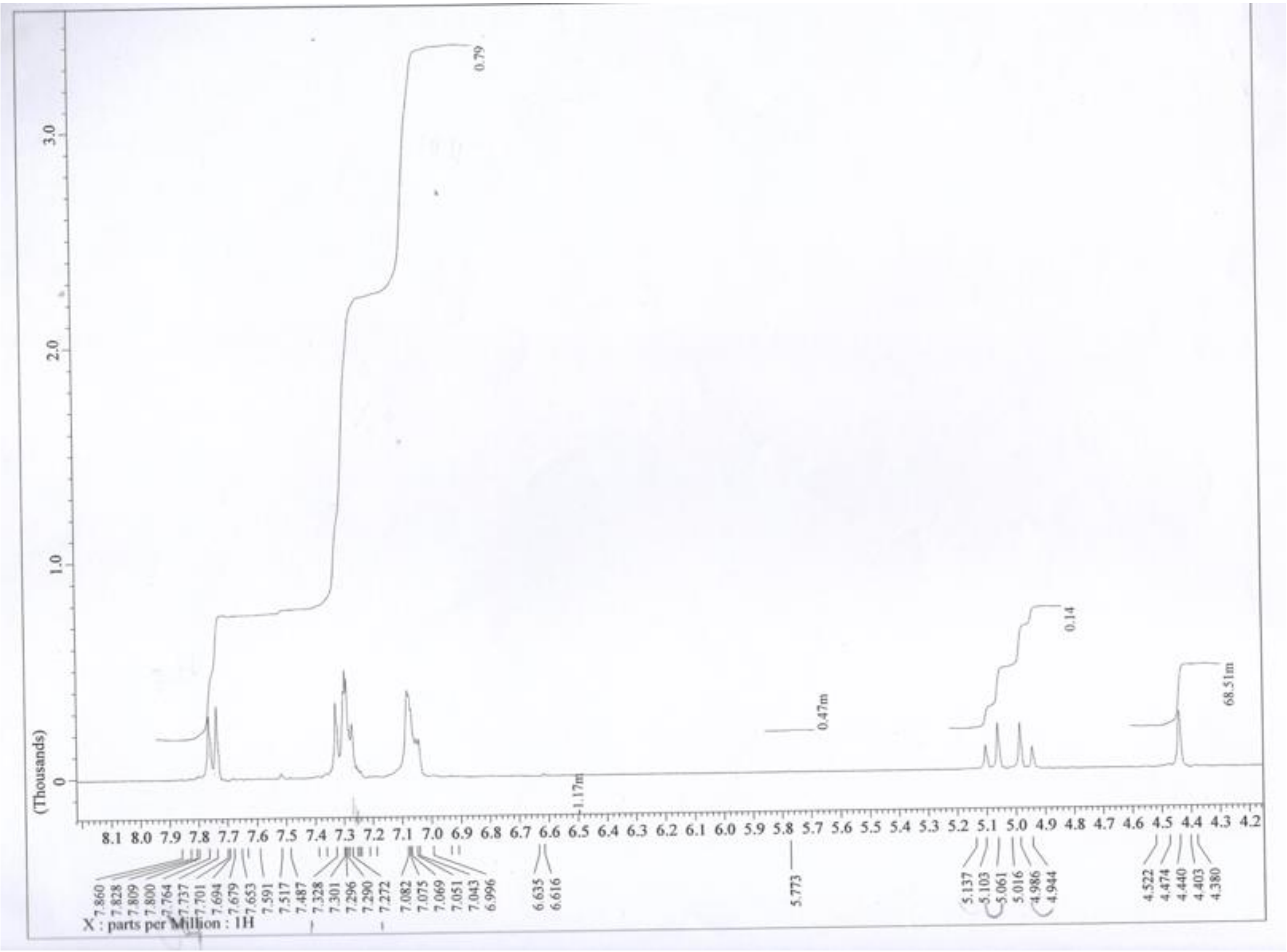

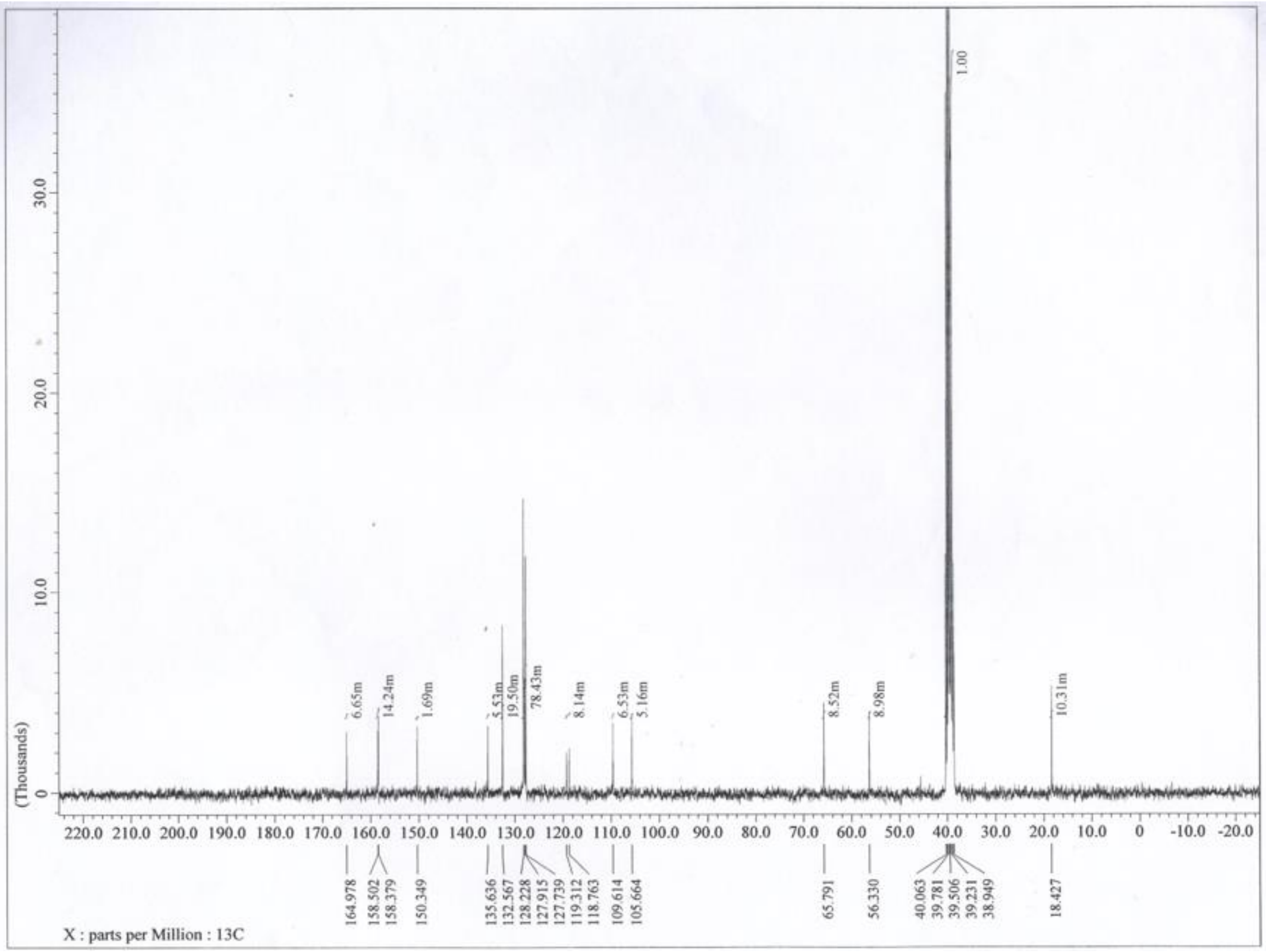
(Thousands)
X : parts per Million : 13C
6.65m
14.24m
1.69m
5.53m
19.50m
78.43m
8.14m
6.53m
5.16m
8.52m
8.98m
1.00
10.31m
164.978
158.502
158.379
150.349
135.636
132.567
128.228
127.915
127.739
119.312
118.763
109.614
105.664
65.791
56.330
40.063
39.781
39.506
39.231
38.949
18.427

Line#:1 R.Time:3.1(Scan#:371)
MassPeaks:228
RawMode:Single 3.1(371) BasePeak:91(2478377)
BG Mode:None Group 1 - Event 1

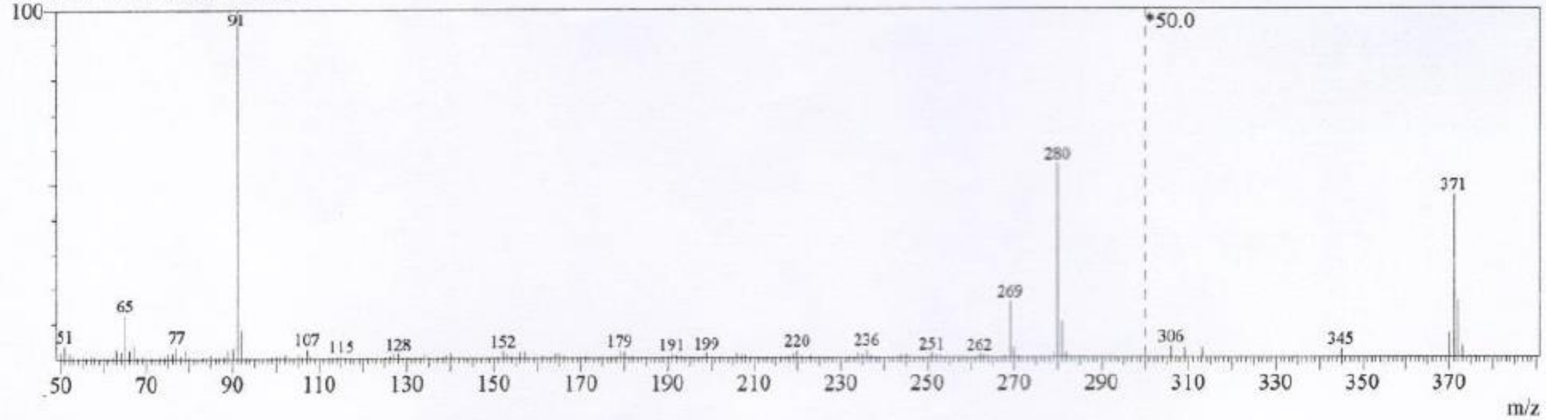

Mass Table
Line#:1 R.Time:3.1(Scan#:371)
MassPeaks:228
RawMode:Single 3.1(371) BasePeak:91(2478377)
BG Mode:None Group 1 - Event 1

#	m/z	Abs. In	Rel. Int.	#	m/z	Abs. In	Rel. Int.	#	m/z	Abs. In	Rel. Int.
1	50.00	38130	1.54	4	53.00	16158	0.65	7	56.05	7191	0.29
2	51.00	90305	3.64	5	54.05	7095	0.29	8	57.05	22025	0.89
3	52.00	35161	1.42	6	55.00	24422	0.99	9	58.00	13943	0.56

1 / 3

#	m/z	Abs. In	Rel. Int.	#	m/z	Abs. In	Rel. Int.	#	m/z	Abs. In	Rel. Int.
10	59.05	4233	0.17	79	128.05	29855	1.20	148	201.95	1549	0.06
11	60.00	9758	0.39	80	129.05	10865	0.44	149	203.00	1817	0.07
12	61.00	7762	0.31	81	130.05	5436	0.22	150	203.95	1597	0.06
13	62.05	17523	0.71	82	131.10	3289	0.13	151	205.05	2664	0.11
14	63.00	62614	2.53	83	132.10	2337	0.09	152	205.95	27356	1.10
15	64.05	40950	1.65	84	133.15	5656	0.23	153	206.95	19789	0.80
16	65.00	305596	12.33	85	134.05	24770	1.00	154	208.00	15678	0.63
17	66.00	56799	2.29	86	135.05	5552	0.22	155	209.00	4775	0.19
18	67.00	95281	3.84	87	136.05	1429	0.06	156	209.95	2530	0.10
19	68.00	11681	0.47	88	137.05	4179	0.17	157	211.00	1478	0.06
20	69.05	14357	0.58	89	138.05	10194	0.41	158	213.05	1632	0.07
21	70.05	4874	0.20	90	139.05	12782	0.52	159	214.05	1195	0.05
22	71.05	11128	0.45	91	140.05	27854	1.12	160	215.05	1061	0.04
23	72.05	1399	0.06	92	141.05	7218	0.29	161	216.00	5073	0.20
24	73.00	10897	0.44	93	142.05	3749	0.15	162	217.00	5772	0.23
25	74.00	14236	0.57	94	143.05	2910	0.12	163	217.95	4473	0.18
26	75.00	34425	1.39	95	144.05	1102	0.04	164	218.95	23479	0.95
27	76.00	32572	1.31	96	145.05	1339	0.05	165	219.95	51019	2.06
28	77.00	82271	3.32	97	146.05	1086	0.04	166	220.95	10480	0.42
29	78.05	20835	0.84	98	147.10	1241	0.05	167	221.95	3876	0.16
30	79.05	60329	2.43	99	149.05	3989	0.16	168	223.00	12751	0.51
31	80.05	7529	0.30	100	150.00	3198	0.13	169	224.00	3842	0.16
32	81.05	5811	0.23	101	151.05	6733	0.27	170	225.00	2286	0.09
33	82.10	3534	0.14	102	152.00	46681	1.88	171	226.00	1322	0.05
34	83.05	9279	0.37	103	153.00	25350	1.02	172	227.05	1761	0.07
35	84.05	7153	0.29	104	154.00	12154	0.49	173	228.05	1101	0.04
36	85.05	21024	0.85	105	155.05	6175	0.25	174	229.95	1398	0.06
37	86.00	5239	0.21	106	156.00	35439	1.43	175	233.05	2348	0.09
38	87.00	10204	0.41	107	157.00	42956	1.73	176	233.95	29689	1.20
39	88.05	13110	0.53	108	158.00	5332	0.22	177	235.00	18716	0.76
40	89.00	56270	2.27	109	159.00	1391	0.06	178	236.00	56961	2.30
41	90.05	78869	3.18	110	160.05	1647	0.07	179	237.00	12006	0.48
42	91.05	247837	100.00	111	161.00	13318	0.54	180	238.00	3306	0.13
43	92.05	196570	7.93	112	161.95	3669	0.15	181	239.10	2175	0.09
44	93.05	9655	0.39	113	163.05	5730	0.23	182	240.00	1322	0.05
45	94.05	2433	0.10	114	164.00	29070	1.17	183	241.05	1253	0.05
46	95.05	5393	0.22	115	165.00	26647	1.08	184	242.05	1037	0.04
47	96.10	3441	0.14	116	166.00	16962	0.68	185	243.95	16032	0.65
48	97.05	9011	0.36	117	167.00	6946	0.28	186	244.95	20742	0.84
49	98.05	6367	0.26	118	168.00	5490	0.22	187	245.95	4612	0.19
50	99.05	11988	0.48	119	169.00	3638	0.15	188	247.00	1407	0.06
51	100.00	14945	0.60	120	170.00	6746	0.27	189	248.00	1623	0.07
52	101.05	22215	0.90	121	171.00	10634	0.43	190	250.05	1394	0.06
53	102.00	25727	1.04	122	172.00	8382	0.34	191	251.00	28382	1.15
54	103.00	9177	0.37	123	173.00	1846	0.07	192	252.00	15634	0.63
55	104.05	4460	0.18	124	173.95	2095	0.08	193	253.00	13044	0.53
56	105.05	21782	0.88	125	176.00	1689	0.07	194	254.00	3794	0.15
57	106.05	11044	0.45	126	177.00	4382	0.18	195	255.05	1909	0.08
58	107.05	67138	2.71	127	178.00	10804	0.44	196	256.05	1701	0.07
59	108.05	18537	0.75	128	178.95	49979	2.02	197	257.05	1575	0.06
60	109.10	4534	0.18	129	180.00	40721	1.64	198	258.00	2345	0.09
61	110.10	2249	0.09	130	181.00	11193	0.45	199	261.05	1215	0.05
62	111.05	5369	0.22	131	182.00	10972	0.44	200	262.00	20161	0.81
63	112.10	5215	0.21	132	183.00	2996	0.12	201	263.00	15046	0.61
64	113.05	9334	0.38	133	183.95	3077	0.12	202	264.00	9580	0.39
65	114.05	7866	0.32	134	185.00	2079	0.08	203	265.00	2270	0.09
66	115.05	14255	0.58	135	189.00	4969	0.20	204	266.00	1180	0.05
67	116.05	8264	0.33	136	190.00	5022	0.20	205	267.05	1366	0.06
68	117.10	4252	0.17	137	190.95	25489	1.03	206	268.05	6182	0.25
69	118.05	3426	0.14	138	191.95	16486	0.67	207	269.05	396493	16.00
70	119.05	3367	0.14	139	193.00	14302	0.58	208	270.00	70511	2.85
71	120.05	1690	0.07	140	193.95	6698	0.27	209	271.00	9028	0.36
72	121.05	2251	0.09	141	195.00	5281	0.21	210	272.00	1658	0.07
73	122.05	2445	0.10	142	195.95	2918	0.12	211	278.05	2796	0.11
74	123.05	2798	0.11	143	197.05	1444	0.06	212	279.05	23573	0.95
75	124.05	4566	0.18	144	198.05	5118	0.21	213	280.00	137926	55.65
76	125.05	9431	0.38	145	198.95	31053	1.25	214	281.00	255044	10.29
77	126.05	13440	0.54	146	199.95	4537	0.18	215	281.95	32319	1.30
78	127.05	26923	1.09	147	201.00	1226	0.05	216	283.00	3695	0.15

#	m/z	Abs. Int	Rel. Int.	#	m/z	Abs. Int	Rel. Int.	#	m/z	Abs. Int	Rel. Int.
217	284.05	1154	0.05	221	306.00	1534	0.06	225	370.05	3319	0.13
218	285.05	2987	0.12	222	309.00	1404	0.06	226	371.00	23178	0.94
219	286.05	1456	0.06	223	313.15	1359	0.05	227	372.00	8198	0.33
220	287.00	1079	0.04	224	345.00	1164	0.05	228	373.05	1594	0.06

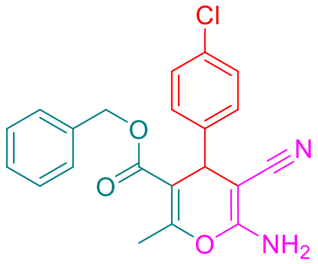

Compound 4l

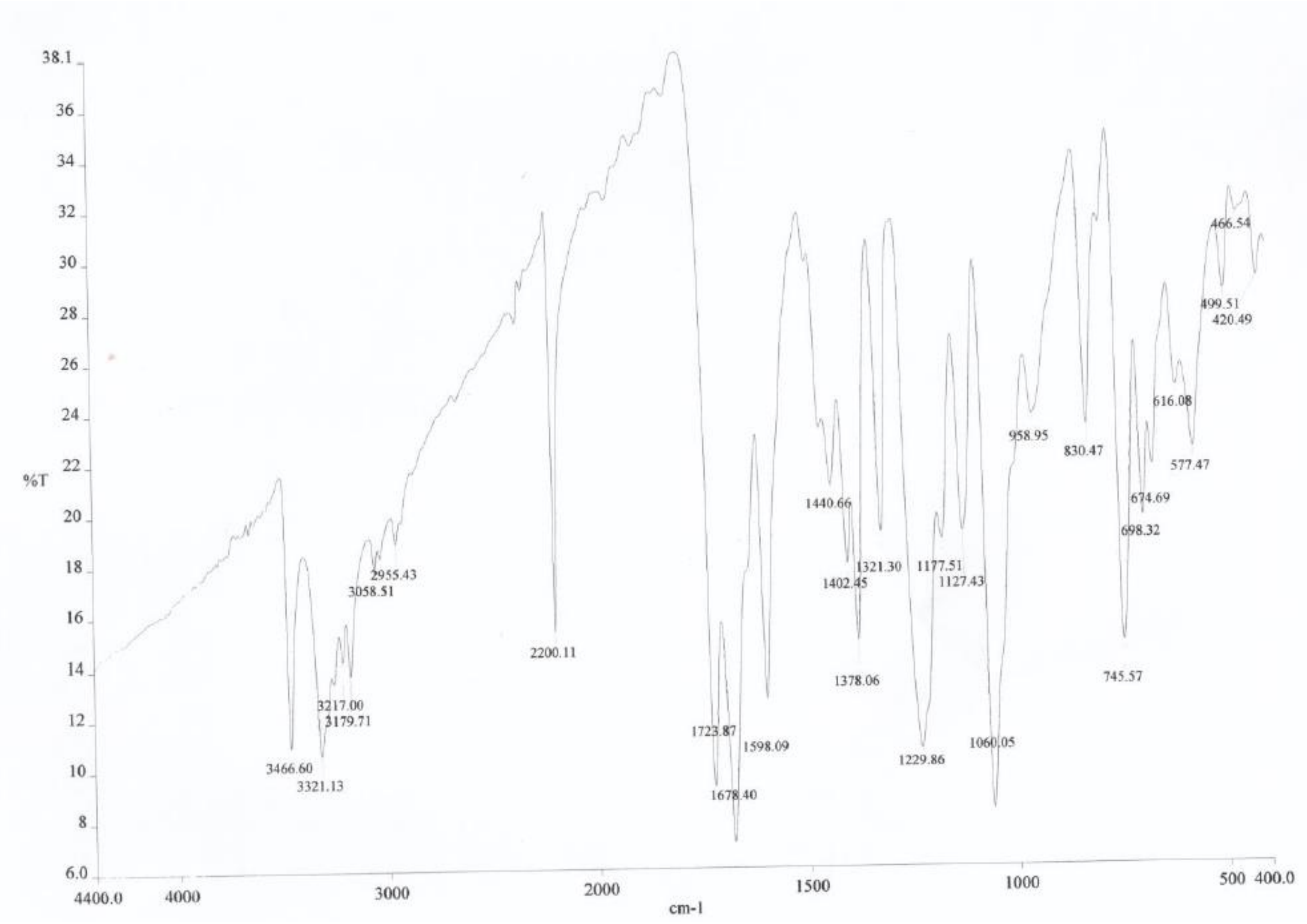
38.1
36
34
32
30
28
26
24
22
%T
20
18
16
14
12
10
8
6.0
4400.0
4000
3000
2000
1500
1000
500
400.0
cm-1
3466.60
3321.13
3217.00
3179.71
3058.51
2955.43
2200.11
1723.87
1678.40
1598.09
1440.66
1402.45
1378.06
1321.30
1229.86
1177.51
1127.43
1060.05
958.95
830.47
745.57
698.32
674.69
616.08
577.47
499.51
466.54
420.49

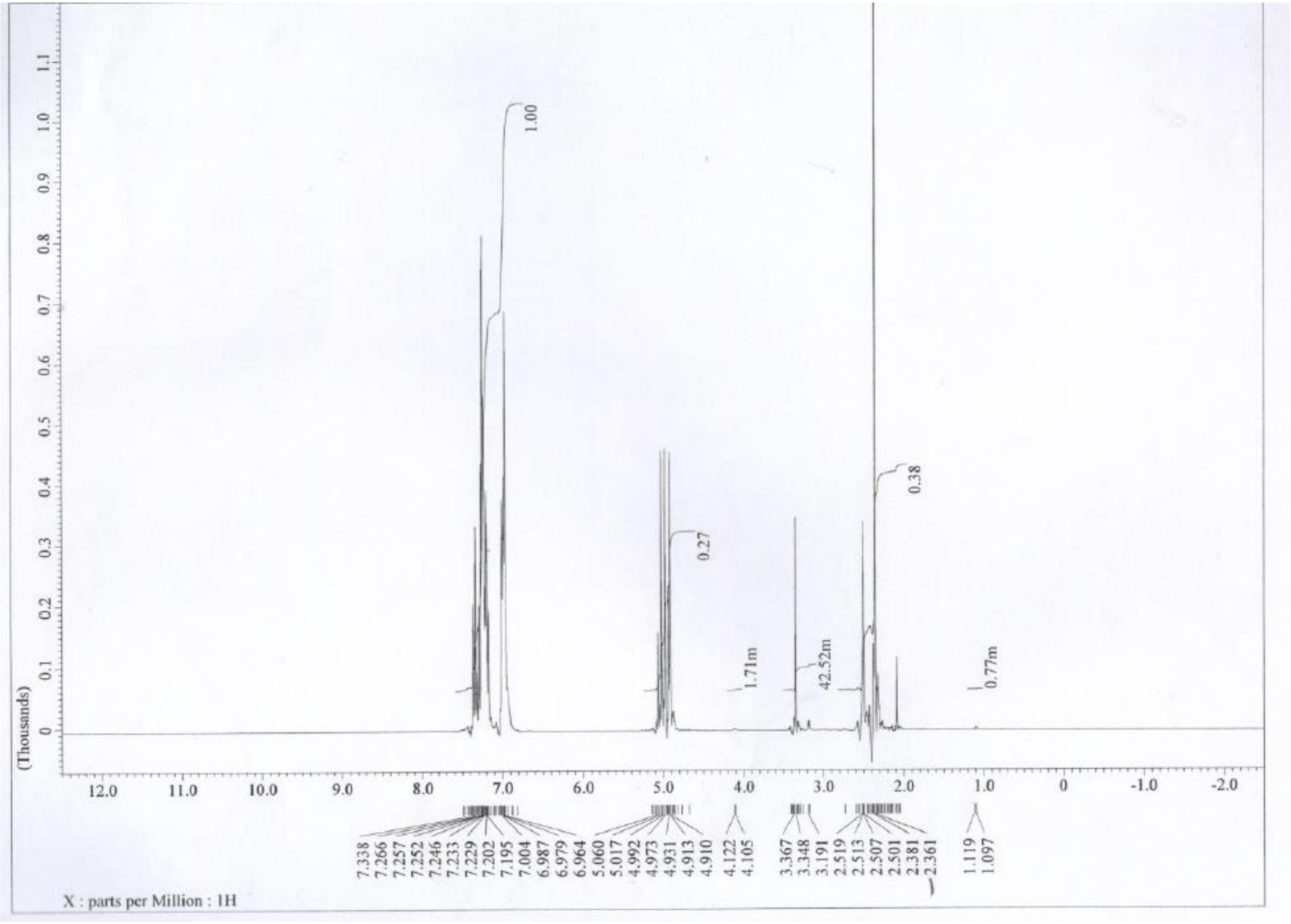
(Thousands)
X : parts per Million : 1H
1.00
0.27
1.71m
42.52m
0.38
0.77m
7.338
7.266
7.257
7.252
7.246
7.233
7.229
7.202
7.195
7.004
6.987
6.979
6.964
5.060
5.017
4.992
4.973
4.931
4.913
4.910
4.122
4.105
3.367
3.348
3.191
2.519
2.513
2.507
2.501
2.381
2.361
1.119
1.097

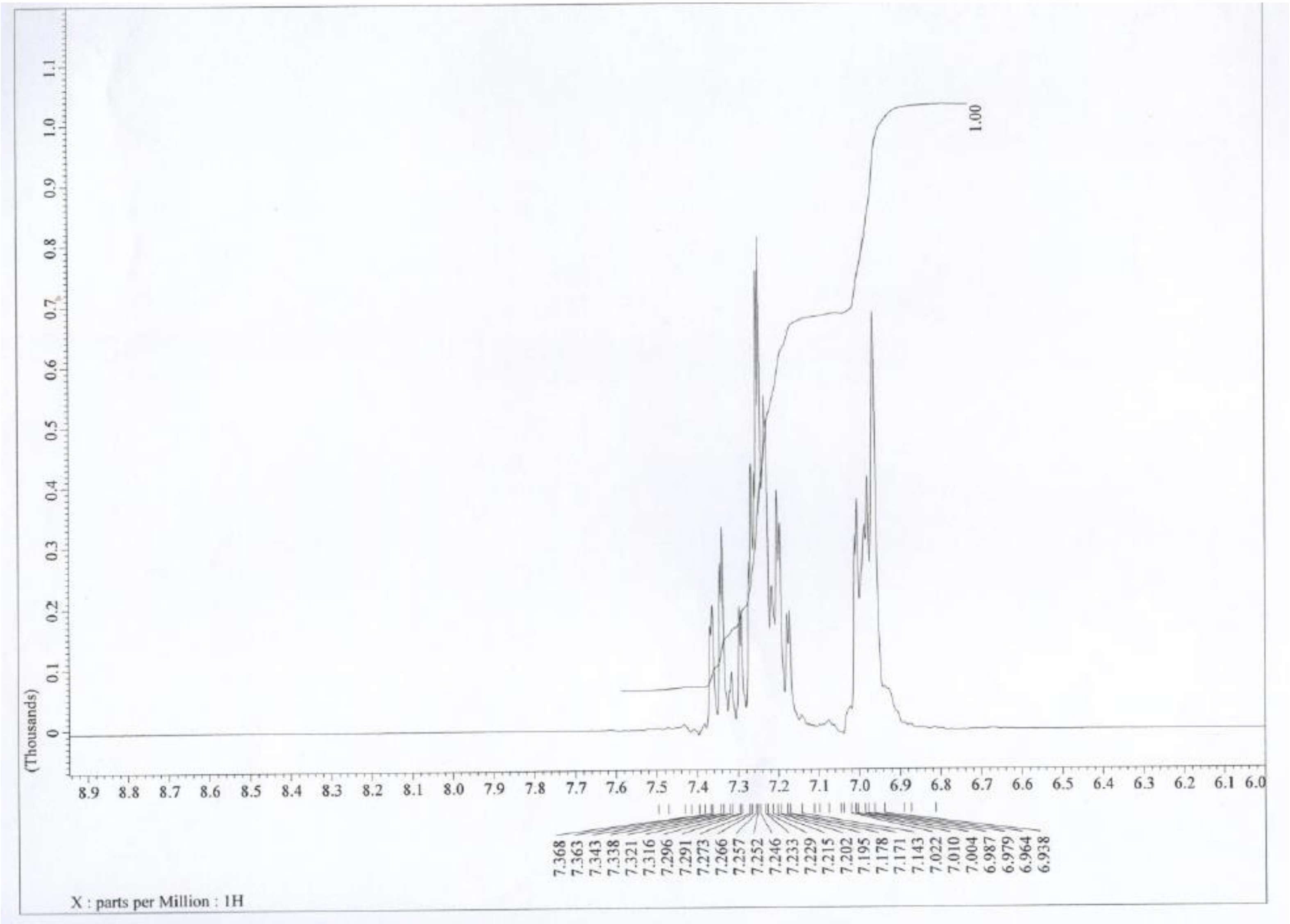

(Thousands)
X : parts per Million : 1H
1.00
7.368
7.363
7.343
7.338
7.321
7.316
7.296
7.291
7.273
7.266
7.257
7.252
7.246
7.233
7.229
7.215
7.202
7.195
7.178
7.171
7.143
7.022
7.010
7.004
6.987
6.979
6.964
6.938

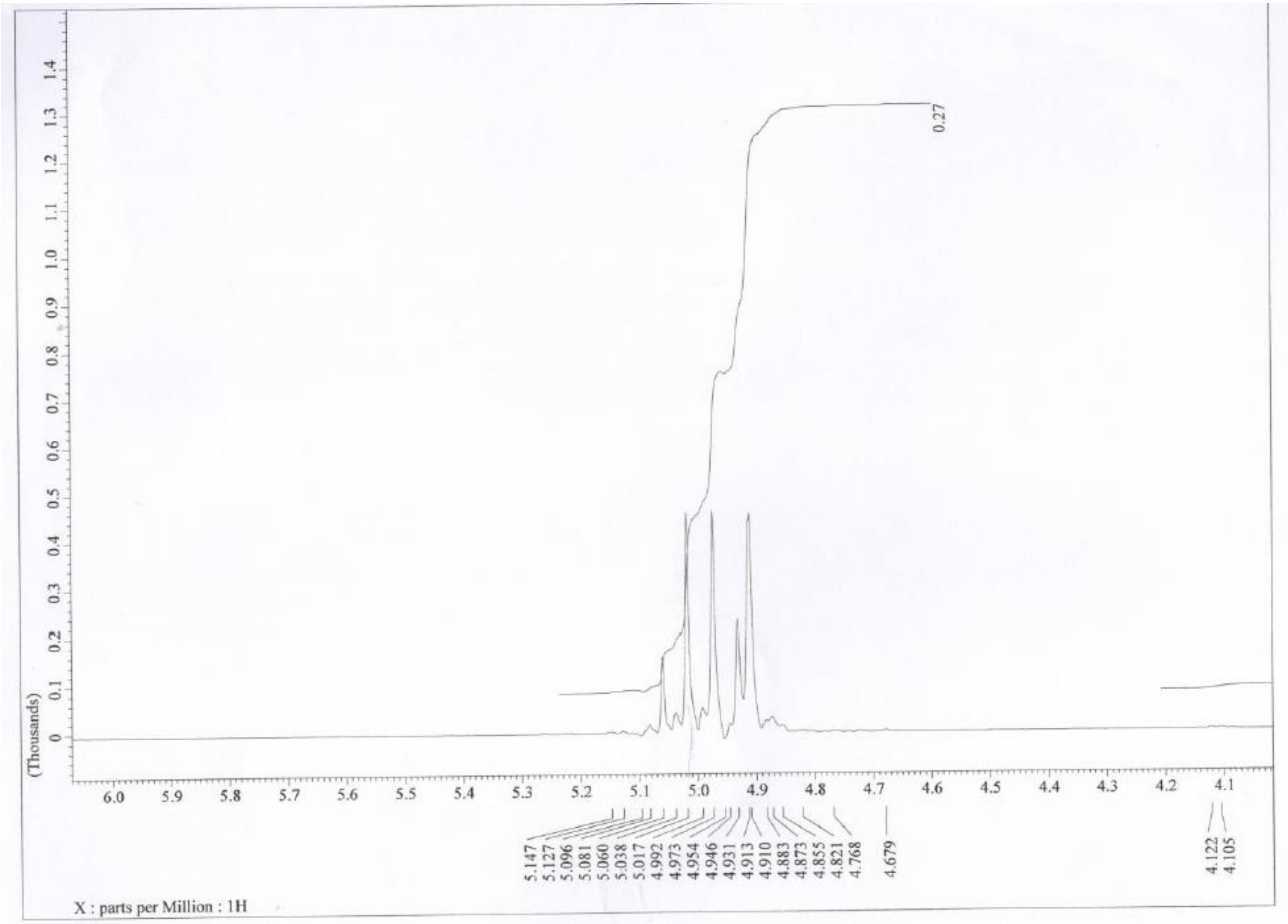

(Thousands)
0.27
X : parts per Million : 1H
5.147
5.127
5.096
5.081
5.060
5.038
5.017
4.992
4.973
4.954
4.946
4.931
4.913
4.910
4.883
4.873
4.855
4.821
4.768
4.679
4.122
4.105

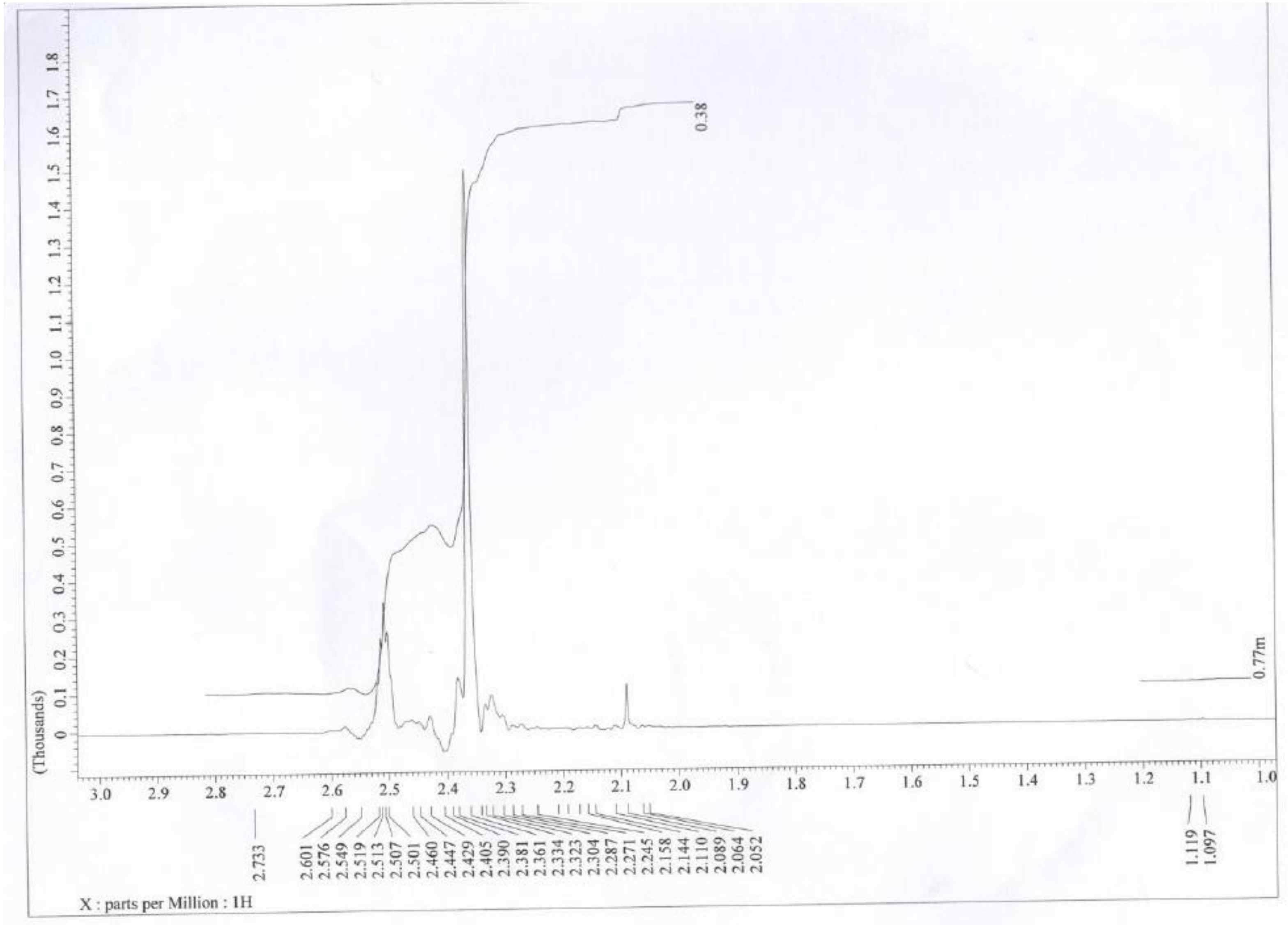

(Thousands)
0.38
0.77m
2.733
2.601
2.576
2.549
2.519
2.513
2.507
2.501
2.460
2.447
2.429
2.405
2.390
2.381
2.361
2.334
2.323
2.304
2.287
2.271
2.245
2.158
2.144
2.110
2.089
2.064
2.052
1.119
1.097
X : parts per Million : 1H

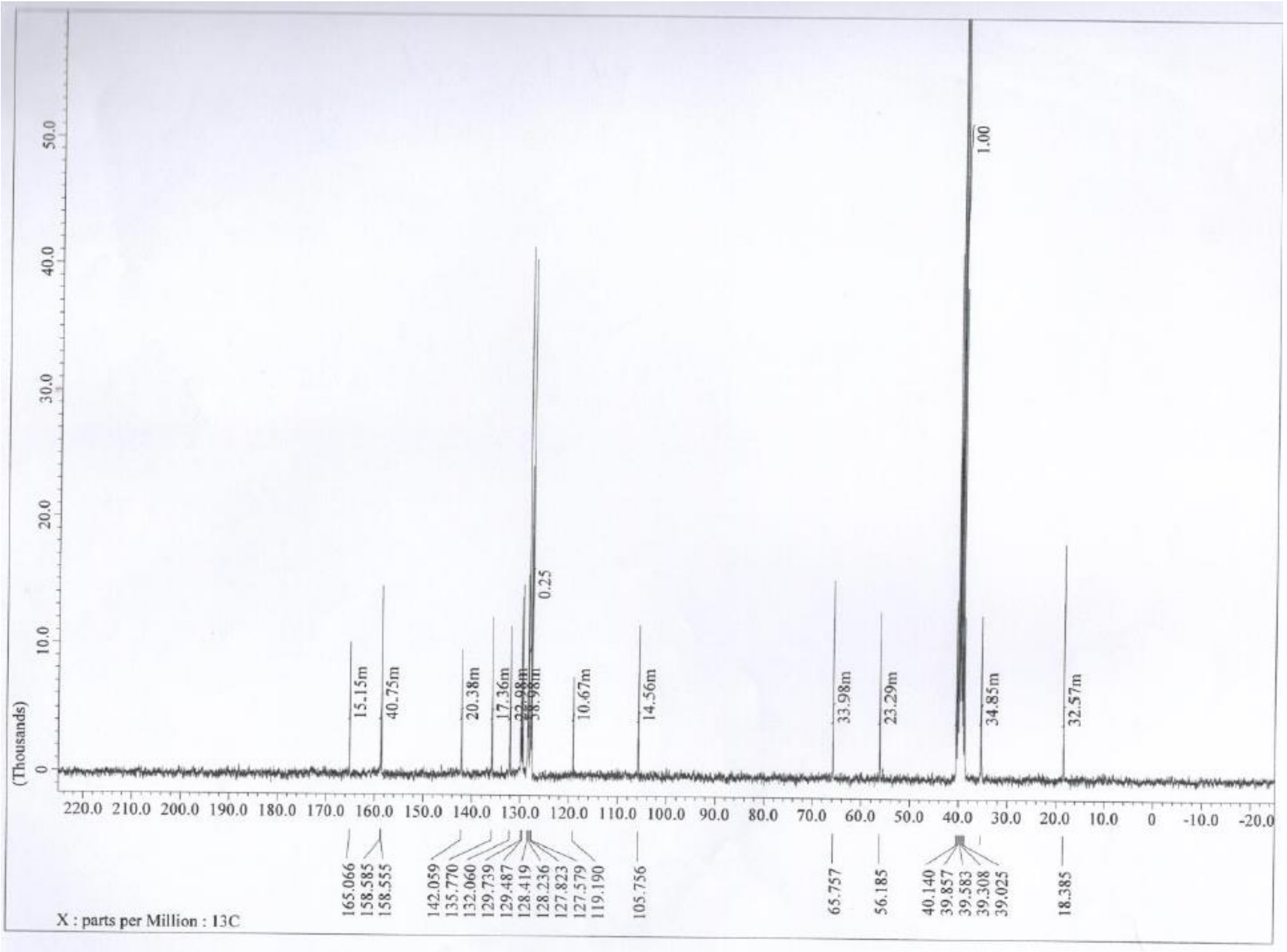
(Thousands)
X : parts per Million : 13C
165.066
158.585
158.555
142.059
135.770
132.060
129.739
129.487
128.419
128.236
127.823
127.579
119.190
105.756
65.757
56.185
40.140
39.857
39.583
39.308
39.025
18.385

Line#:2 R.Time:3.2(Scan#:382)
MassPeaks:240
RawMode:Single 3.2(382) BasePeak:91(4538062)
BG Mode:None Group 1 - Event 1

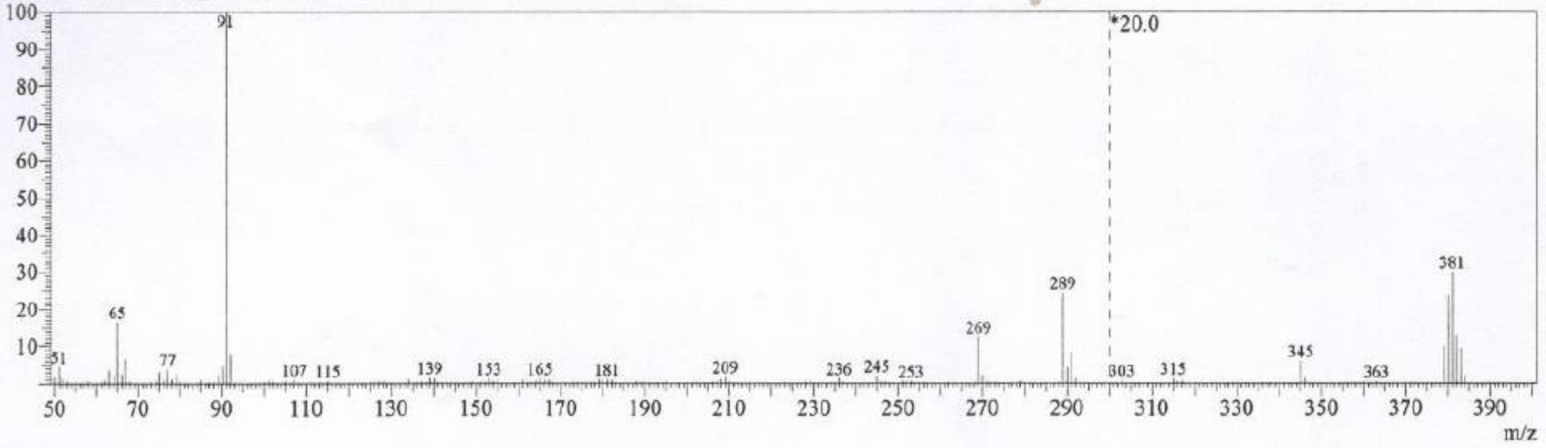

Mass Table
Line#:1 R.Time:1.8(Scan#:213)
MassPeaks:226
RawMode:Single 1.8(213) BasePeak:91(1976876)
BG Mode:None Group 1 - Event 1

#	m/z	Abs. In	Rel. Int.	#	m/z	Abs. In	Rel. Int.	#	m/z	Abs. In	Rel. Int.
1	50.00	32201	1.63	46	95.05	3421	0.17	91	141.05	9319	0.47
2	51.00	78199	3.96	47	96.10	2207	0.11	92	142.10	2914	0.15
3	52.00	25655	1.30	48	97.05	8018	0.41	93	143.10	4522	0.23
4	53.00	12378	0.63	49	98.05	4261	0.22	94	144.10	2986	0.15
5	54.05	5763	0.29	50	99.05	13802	0.70	95	145.05	5046	0.26
6	55.00	15807	0.80	51	100.05	10987	0.56	96	146.05	1146	0.06
7	56.05	4893	0.25	52	101.05	25612	1.30	97	147.05	1933	0.10
8	57.05	15289	0.77	53	102.05	16296	0.82	98	148.05	1572	0.08
9	58.00	9228	0.47	54	103.05	3612	0.18	99	149.00	12615	0.64
10	59.00	2566	0.13	55	104.15	2387	0.12	100	150.05	3624	0.18
11	60.00	3598	0.18	56	105.05	17020	0.86	101	151.05	5877	0.30
12	61.00	5314	0.27	57	106.10	8306	0.42	102	152.05	7992	0.40
13	62.05	14311	0.72	58	107.05	27670	1.40	103	153.05	36006	1.82
14	63.00	57111	2.89	59	108.05	8355	0.42	104	154.05	12531	0.63
15	64.05	30562	1.55	60	109.05	3376	0.17	105	155.05	9082	0.46
16	65.00	247215	12.51	61	110.05	2741	0.14	106	156.05	2451	0.12
17	66.05	35717	1.81	62	111.05	14959	0.76	107	157.05	1518	0.08
18	67.00	92036	4.66	63	112.05	7014	0.35	108	160.05	1191	0.06
19	68.00	10015	0.51	64	113.05	14534	0.74	109	161.00	18938	0.96
20	69.05	9983	0.50	65	114.05	8599	0.43	110	162.00	8660	0.44
21	70.05	3228	0.16	66	115.05	19148	0.97	111	163.00	9638	0.49
22	71.05	8292	0.42	67	116.10	5874	0.30	112	164.00	16825	0.85
23	72.05	1336	0.07	68	117.10	3377	0.17	113	164.95	26320	1.33
24	73.00	7716	0.39	69	118.05	2907	0.15	114	166.00	18545	0.94
25	74.05	15676	0.79	70	119.05	2894	0.15	115	167.00	21142	1.07
26	75.00	56441	2.86	71	121.05	1604	0.08	116	168.00	8537	0.43
27	76.05	20370	1.03	72	122.05	2006	0.10	117	169.00	2230	0.11
28	77.05	68758	3.48	73	123.05	3018	0.15	118	170.05	1032	0.05
29	78.05	17958	0.91	74	124.05	2740	0.14	119	171.00	5887	0.30
30	79.05	43343	2.19	75	125.05	9179	0.46	120	172.05	1285	0.07
31	80.05	6227	0.31	76	126.05	18835	0.95	121	173.00	10479	0.53
32	81.05	3941	0.20	77	127.05	23148	1.17	122	174.00	8868	0.45
33	82.10	2570	0.13	78	128.10	17452	0.88	123	175.00	7954	0.40
34	83.10	6105	0.31	79	129.10	5956	0.30	124	175.95	4988	0.25
35	84.05	5049	0.26	80	130.10	1746	0.09	125	177.00	7686	0.39
36	85.00	21319	1.08	81	131.10	1382	0.07	126	178.00	5894	0.30
37	86.00	5599	0.28	82	132.10	1274	0.06	127	179.00	14127	0.71
38	87.00	8794	0.44	83	133.15	5478	0.28	128	180.00	24593	1.24
39	88.05	8953	0.45	84	134.10	22330	1.13	129	181.00	20444	1.03
40	89.05	46953	2.38	85	135.05	5215	0.26	130	182.00	17866	0.90
41	90.05	54701	2.77	86	136.05	7346	0.37	131	183.00	6953	0.35
42	91.05	197687	100.00	87	137.05	14311	0.72	132	184.00	2734	0.14
43	92.05	156782	7.93	88	138.05	14382	0.73	133	185.05	1263	0.06
44	93.05	7648	0.39	89	139.05	36540	1.85	134	187.00	2614	0.13
45	94.05	2013	0.10	90	140.05	30360	1.54	135	187.95	12865	0.65

#	m/z	Abs. In	Rel. Int.	#	m/z	Abs. In	Rel. Int.	#	m/z	Abs. In	Rel. Int.
136	189.00	9553	0.48	167	221.00	1260	0.06	198	256.00	2937	0.15
137	189.95	8100	0.41	168	223.05	11103	0.56	199	257.05	1912	0.10
138	191.00	5964	0.30	169	224.00	3199	0.16	200	261.05	2849	0.14
139	192.00	5844	0.30	170	225.00	3134	0.16	201	262.00	6978	0.35
140	193.00	4593	0.23	171	226.00	2244	0.11	202	263.00	2242	0.11
141	194.05	3676	0.19	172	227.05	2527	0.13	203	264.00	2529	0.13
142	195.00	13518	0.68	173	228.00	12326	0.62	204	268.15	10429	0.53
143	196.00	3114	0.16	174	228.95	16494	0.83	205	269.05	429561	21.73
144	197.05	1328	0.07	175	229.95	7881	0.40	206	270.05	75120	3.80
145	197.95	1593	0.08	176	230.95	7271	0.37	207	271.00	16881	0.85
146	199.05	1317	0.07	177	232.00	1860	0.09	208	272.00	11702	0.59
147	199.95	7662	0.39	178	233.05	2092	0.11	209	273.00	9337	0.47
148	201.00	5606	0.28	179	234.00	11143	0.56	210	274.00	4671	0.24
149	202.00	8769	0.44	180	235.05	4660	0.24	211	275.00	2820	0.14
150	202.95	5893	0.30	181	236.00	48682	2.46	212	279.05	13011	0.66
151	203.95	3740	0.19	182	237.00	25834	1.31	213	280.05	2772	0.14
152	205.05	2911	0.15	183	238.00	4622	0.23	214	288.05	12229	0.62
153	206.00	14089	0.71	184	239.05	2084	0.11	215	289.00	904460	45.75
154	207.00	10484	0.53	185	240.05	1476	0.07	216	290.00	152519	7.72
155	208.00	25386	1.28	186	243.00	4743	0.24	217	291.00	303908	15.37
156	209.00	39159	1.98	187	244.05	8460	0.43	218	292.00	49985	2.53
157	210.00	10812	0.55	188	245.00	64330	3.25	219	292.95	6032	0.31
158	211.00	5169	0.26	189	246.00	13092	0.66	220	345.05	9213	0.47
159	212.00	2177	0.11	190	247.00	23327	1.18	221	346.05	2271	0.11
160	213.00	1082	0.05	191	248.00	3674	0.19	222	379.05	2892	0.15
161	215.00	3820	0.19	192	249.05	1235	0.06	223	380.00	30342	1.53
162	216.00	4743	0.24	193	251.05	24110	1.22	224	381.05	9439	0.48
163	216.95	3374	0.17	194	252.05	12294	0.62	225	382.00	11164	0.56
164	217.95	2640	0.13	195	253.00	14918	0.75	226	383.00	2874	0.15
165	219.00	2314	0.12	196	254.00	7844	0.40				
166	220.00	1119	0.06	197	255.00	4310	0.22				
